# A critical review of degradation pathways and stability engineering for high-efficiency non-fullerene acceptor-based organic solar cells *via* multiscale computational approaches

**DOI:** 10.1039/d6ra08413c

**Published:** 2026-10-28

**Authors:** Qurat-Ul-Ain, Abdul Majid, Muhammad Tayyab Raza, Sheraz Ahmad

**Affiliations:** a Department of Chemistry, Government College University Lahore Lahore Pakistan quratulainsandhu32@gmail.com; b Department of Physics, Hafiz Hayat Campus, University of Gujrat Gujrat Pakistan; c Department of Physics, Gachon University 1342 Seongnam-daero, Sujeong-gu Seongnam-si Gyeonggi-do South Korea ali1214@gachon.ac.kr; d Dipartimento Di Architettura E Disegno Industriale Via San Lorenzo – Abazia di San Lorenzo 81031-Aversa CE Italy

## Abstract

The emergence of non-fullerene acceptors (NFAs) has significantly transformed the landscape of organic solar cells (OSCs) and paved the way for achieving unprecedented power conversion efficiencies (PCEs) and making them suitable for practical photovoltaic applications. Computational techniques, meanwhile, have become essential for the efficient discovery, screening, and optimization of new photovoltaic (PV) materials. This review gives a comprehensive summary of the recent developments in computational design and construction of NFAs with special attention to the use of computational tools in the understanding and enhancement of the structural, electronic and optoelectronic properties of NFAs. In addition, the challenges related to the stability and degradation of NFAs are briefly addressed, with prominence on how computational methods can contribute to understanding these issues. Furthermore, this review comprehensively evaluates the core computational strategies used to probe NFA degradation mechanisms specifically photostability, thermal stress, morphological evolution and oxidative pathways, and offers computational solutions to improve the long-term stability of OSC devices. As the efficiency of OSCs has approached the benchmark values, the attention towards their practical application has risen substantially. Therefore, the importance of computational techniques like density functional theory (DFT), time-dependent DFT (TD-DFT), machine learning (ML) and molecular dynamics (MD) simulations is discussed to highlight the key aspects of NFA performance including charge transport, light harvesting, device stability, and exciton dissociation. Moreover, the role of these computational tools in understanding and predicting the structure–property relationships is described, which helps in the design of new NFA materials with enhanced device performance. Finally, the breakthroughs and future perspectives in the design of NFAs using computational approaches are discussed.

## Abstract 2

The emergence of non-fullerene acceptors has significantly transformed the landscape of organic solar cells and paved the way for achieving unprecedented power conversion efficiencies for practical photovoltaic applications.

## Graphical abstract

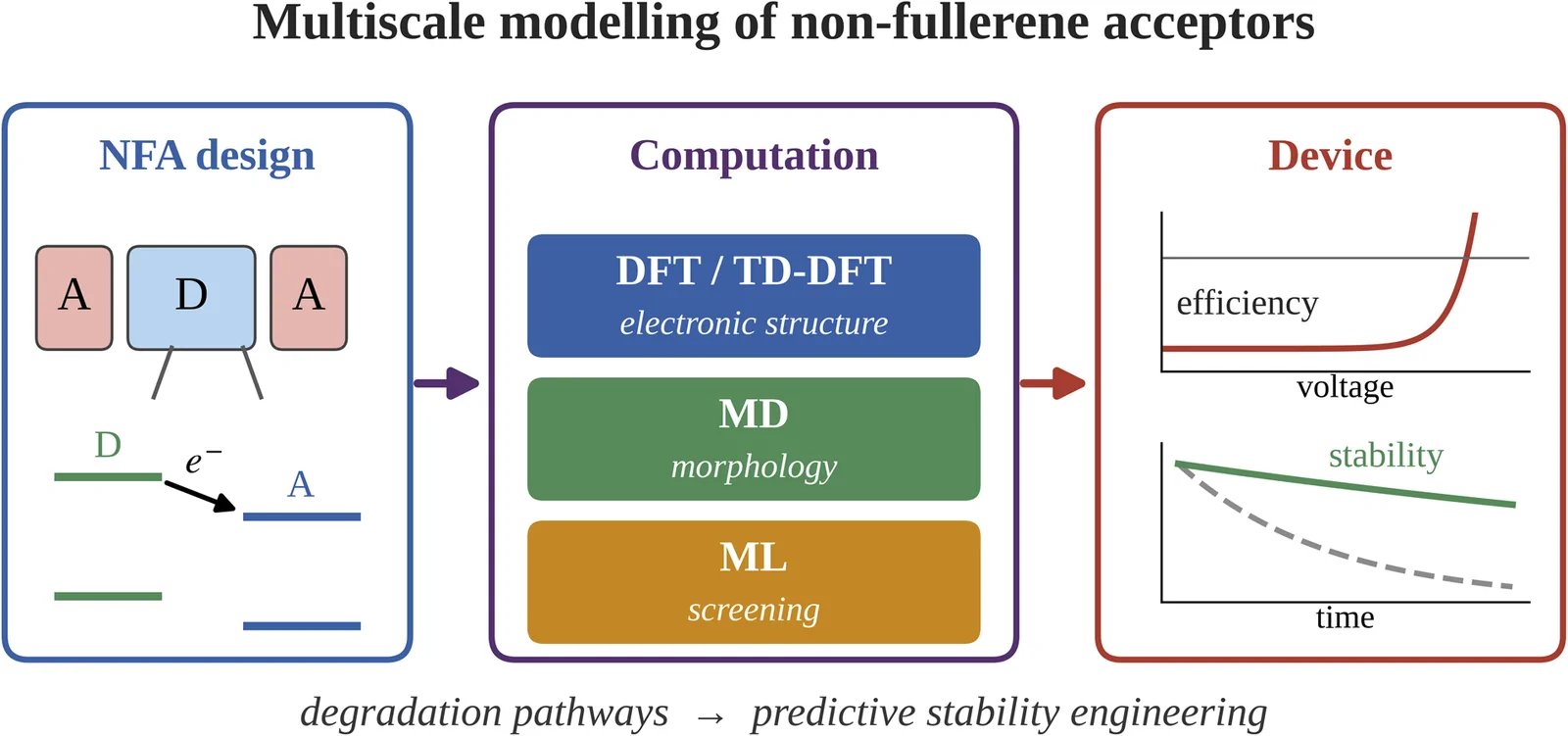

## 1. Introduction

Organic solar cells (OSCs) have become one of the most promising renewable energy technologies due to their low fabrication cost, mechanical flexibility, lightweight feature, optical transparency, and continuously improved photovoltaic performance.^1,2^ The working principle of OSCs is based on the conversion of solar energy to electrical energy *via* a sequence of linked photophysical and photochemical processes in the semiconducting active layer. The history of OSC technology dates back to the 1950s when it was found that organic dyes can exhibit photoconductive properties. These initial studies paved the way for the development and further progress of organic photovoltaic materials.^3^ There are two main architectures of OSC: bilayer (flat heterojunction) and bulk heterojunction (BHJ), as illustrated in Fig. 1. The bilayer architecture consists of two planar interfaces (donor and acceptor layers) arising from the deposition of two planar layers. However, the small interfacial area limits the efficiency of exciton dissociation. In contrast, the most popular BHJ configuration involves mixing donor and acceptor materials in a nanoscale architecture resulting in a bicontinuous structure with a high nanoscale interface area which provides an efficient charge separation pathway. This mixture is considered the most important part of OSCs, because the conversion of solar energy takes place in it.^4,5^

**Fig. 1:**
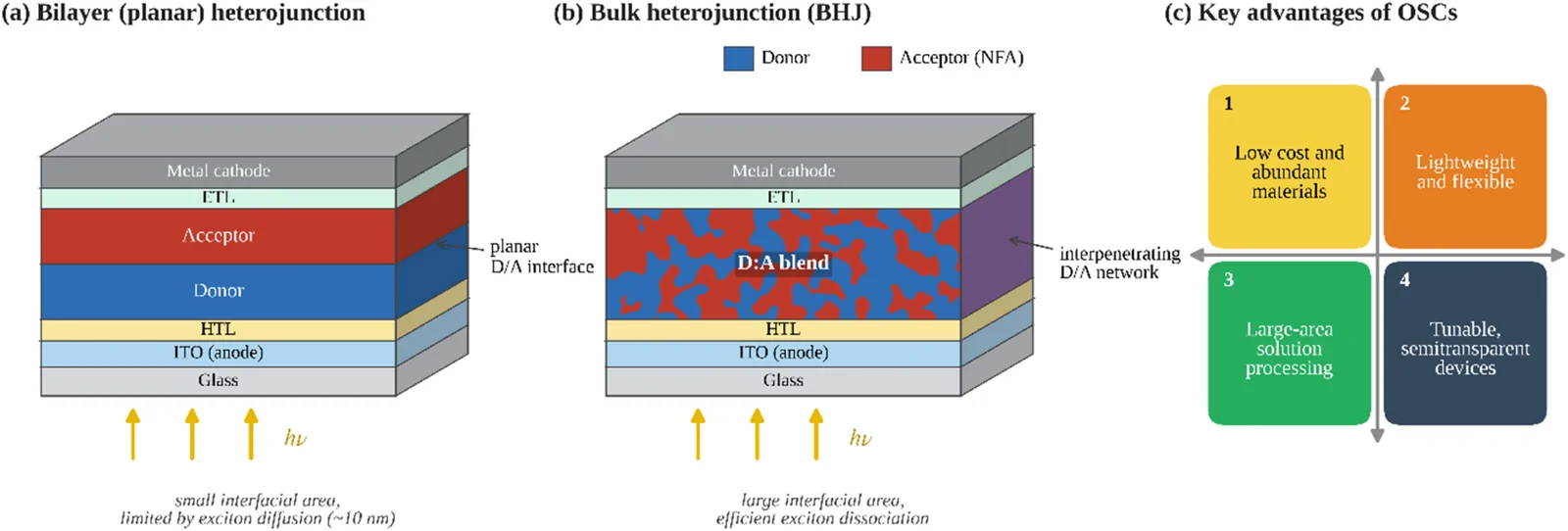
Schematic illustration of OSC architectures: (a) bilayer (planar) heterojunction and (b) bulk heterojunction (BHJ) devices, showing the key layers and their arrangement, and (c) the key advantages of OSCs in four distinct categories.

In OSCs, the process of solar to electrical energy conversion is divided into four basic steps. Firstly, the absorption of a photon creates bound electron–hole pairs (excitons). Secondly, such excitons need to be transported to the donor/acceptor interface within the exciton diffusion length, which is typically in the range of 5–20 nm. Thirdly, the excitons dissociate into free charge carriers at the interface if the energy offset is large enough. Fourthly, the separated electrons and holes pass through the acceptor and donor phases, respectively, and are gathered on the electrodes.^6,7^ Each of these steps has its own challenges, and computational tools can help to address them. There are four important parameters used to quantify the efficiency of OSCs. The short-circuit current density (*J*_sc_) indicates the maximum current density at a voltage of zero and a higher value means higher efficiency of photon harvest. The open-circuit voltage (*V*_oc_) is the maximum voltage difference the device can generate, and a higher *V*_oc_ requires an optimal energy level alignment.

Fill factor (FF) is a measure of the squareness of the current–voltage (*I*–*V*) curve; higher values signify that more charges are extracted from the material and that there is less recombination loss. The power conversion efficiency (PCE) is the overall device efficiency which is equal to PCE = (*J*_sc_ × *V*_oc_ × FF)/*P*_in_, where *P*_in_ is the power density of the incident light. The current state-of-the-art single-junction OSC operates with a PCE greater than 20%, and tandem OSC devices have been demonstrated to operate with a PCE greater than 22%. OSCs can be made in a number of different architectures.^8,9^ The conventional or normal structure is composed of transparent conductive oxide, hole transport layer, active layer, electron transport layer, and metal cathode. The inverted architecture inverts this stack, resulting in better stability because the acidic hole transport layer is not present in this version. Multi-junction or tandem devices combine multiple sub-cells absorbing complementary wavelengths, in order to reduce thermalization losses. In terms of active layer compositions, single donor-single acceptor active layer combinations are most frequent. For better absorption and optimal morphology, ternary blends are used, which contain two donors or two acceptors. Planar heterojunctions or bilayer structures are made up of discrete layers of donors and acceptors, but suffer from short exciton diffusion lengths. Pseudo-planar heterojunction structures are made by a layer-by-layer processing with vertical phase separation.^10,11^

In an OSC, the acceptor material is important to accept electrons from the donor material.^12^ Depending on their molecular structure, they can be divided into two groups: fullerene-based acceptors and non-fullerene acceptors (NFAs).^13^ The morphology of the acceptors, along with their good charge transport, high electron affinity (EA) and good phase separation characteristics, made fullerene acceptors, such as PC71BM, indene-C_60_ bis-adduct (ICBA) and PC61BM (Fig. 2), widely used. These materials have been used in combination with appropriate donor materials with PCEs of around 12%.^14^ They have, however, a number of disadvantages including low optical absorption, insufficient tunability of their energy levels, poor stability, and a fixed molecular structure that prevent further enhancements in performance.^15,16^ Furthermore, fullerene aggregation in the active layer may result in undesirable changes to morphology, charge transport and exciton dissociation, which will reduce OSC efficiency.^17^

**Fig. 2:**
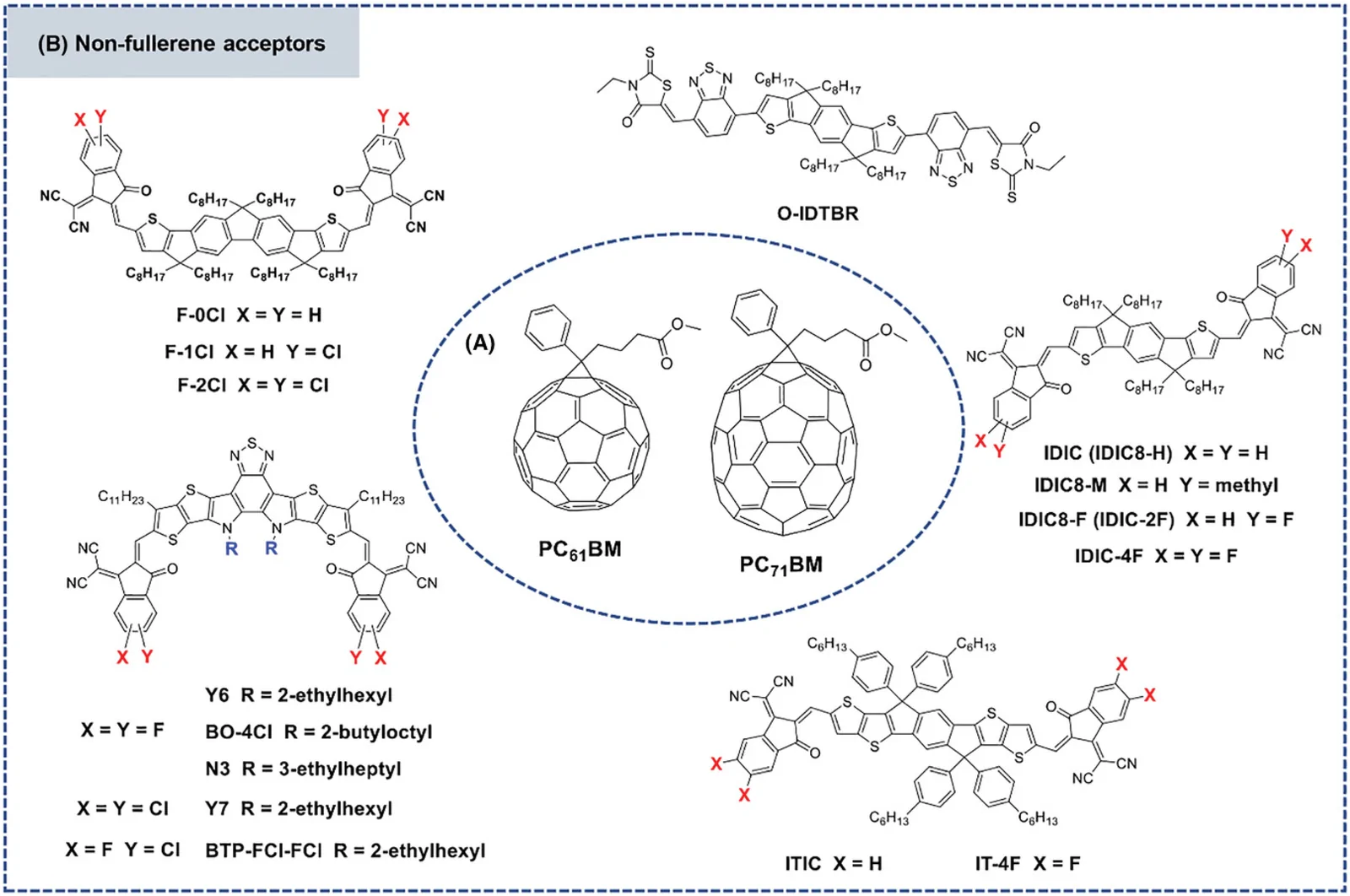
(A) Chemical structures of representative fullerene acceptors PC_61_BM and PC_71_BM, and (B) various nonfullerene acceptors (NFAs), including IDIC and its derivatives (IDIC8-M, IDIC8-F, IDIC-4F), Y-series acceptors (Y6, BO-4Cl, N3, Y7, BTP-FCl-FCl), F-series acceptors (F-OCl, F-1Cl, F-2Cl), and O-IDTBR, which demonstrate synthetic tunability and structural diversity. This figure has been adapted/reproduced from ref. 18 with permission from John Wiley and Sons, copyright 2021.

There are a number of basic drawbacks that have stymied further advances in the field of fullerene acceptors. Optically, they are poorly absorbing in the visible and near-infrared region (*ε* < 5000 M^−1^ cm^−1^) and they can absorb only 30–40% of the solar photons. Electronically, their energy levels are restricted to a rather small window (−3.8 to −4.2 eV) which does not allow optimal alignment with a wide variety of donors.^19^ The recombination losses are high for fullerenes because they have a large exciton diffusion length, and they tend to crystallize into large domains (>100 nm). Synthetically, fullerenes are produced through complex multi-step synthesis (5–8 steps), high production cost (1000–5000 per gram) and limited functionalization positions. In addition, they are prone to degrading upon exposure to light and are very susceptible to oxygen and moisture, and further enhanced by the formation of singlet oxygen. Examples are PC_61_BM, which has high costs, low solubility, and poor thermal stability. PC_71_BM has slightly better absorption properties, but has poor optical properties. ICBA has a higher lowest unoccupied molecular orbital (LUMO) level (−3.74 eV) which leads to higher *V*_oc_, and its electron mobility is lower, but also offers limited derivatization.^16,17,20^

For efficient operation of organic photovoltaics (OPVs), it is important that the donor and acceptor phases form a stable, interpenetrating bicontinuous network which allows continuous pathways for the transport of charges to their respective electrodes. To attain this ideal morphology, it is required that the domain size be comparable to the exciton diffusion length (usually 10–20 nm), that there be no dead ends in the percolation pathway, that there be a high interfacial area for efficient exciton dissociation, and that the morphology be stable under operation conditions.^21^ The morphology is prone to change over time because of the strong aggregation tendency of fullerenes and their poor alignment with donors. The geometry of fullerene molecules is spherical, and the aggregate is also formed in an isotropic manner, which hinders the possibility of control of the domain size. In thermal annealing, the phase separation usually happens at a higher rate than the device performance can be maintained. The electronic structure of fullerenes is completely different from that of NFAs. The fullerenes have a spherical, rigid structure, which is also characterized by a delocalized π-electron system, leading to fixed energy levels and limited tuning ability. In contrast, the push–pull architecture in NFAs (A–D–A or A–DA′D–A) permits the tuning of the highest occupied molecular orbital (HOMO) and LUMO levels independently by molecular engineering. This difference in architecture allows NFAs to have broader absorption spectra, smaller bandgap and improved alignment of energy levels with different donor materials. Fullerenes generally have a LUMO between −3.8 and −4.2 eV, whereas the LUMO of NFAs such as Y6 is as low as −4.0 eV, and its EA and charge transport properties are much better. NFAs offer a unique advantage over fullerenes because they can adjust their energy level through independent side-chain modification, core engineering and terminal group functionalization.^20,22,23^

To address the shortcomings of fullerene acceptors, another type of acceptor materials has been developed, the polymer- and small-molecule-based NFAs.^21,22,24^ The first polymer acceptor based on perylene diimide was reported in 2007 after which it has been used in all-polymer solar cells on the basis of its high electron mobility and broad absorption.^25^ It paved the way for the development of both polymer and small-molecule non-fullerene acceptors. The light harvesting effect in NFAs is significantly greater and there is a broad absorption range into the near-infrared region, which leads to a higher photocurrent generation.^23^ These electronic and optical properties can be readily engineered with a molecular design approach and their solution processability and ease of fabrication make them attractive for low cost, large-scale OSC manufacture. When compared with fullerene acceptors, NFAs exhibit better morphology, smaller energetic disorder, lower charge recombination rate and tunable electron affinity, allowing great progress in OPVs. The OSCs based on polymer donors and the fullerene acceptor PCBM reached a PCE of around 9%. The following studies focused on the design of more complex structures, such as small-molecule acceptor–donor–acceptor (A–D–A) acceptors or fused-ring electron acceptors (FREAs). The concept of FREAs was first reported with a planar fused-ring acceptor (IDIC), which offered an efficiency of 8.71% in as-cast polymer solar cells.^26^ Out of the FREAs, ITIC merged with the donor PTB7-Th delivered a PCE of 6.8% (ref. 27) whereas the A–DA′D–A-type acceptor Y6, when blended with PM6, yielded a PCE of nearly 16%.^28^ To achieve additional improvements in NFA performance, there is a need to understand structure–property relationships and effective molecular design strategies.^29^ First-principles approaches, such as density functional theory (DFT), along with molecular dynamics (MD) and machine learning (ML), have become more popular for predicting molecular structures, electronic properties and intermolecular interactions prior to experimental synthesis.^30^

First-principles methods and ML are now well established tools for predicting and discovering materials' structures. Of these, DFT is commonly employed to study frontier energy levels, charge distribution and intermolecular interactions of NFAs as shown in Fig. 3.^31^ It also facilitates the accurate analysis of the density of states (DOS), frontier molecular orbital, HOMO/LUMO energy levels and electronic structures of organic semiconductors.^32^ Due to their outstanding optoelectronic properties, π-conjugated molecular systems have garnered a lot of attention in the field of organic electronics.^33^ A number of exchange–correlation functionals have been used widely to optimize structures and to predict the properties of NFAs, which include ωB97XD, CAM-B3LYP, MPW1PW91, and B3LYP. Optimization of the IDTT-BO-MN acceptor was performed in Gaussian at the B3LYP/6-31G level to study the structure and electronic properties of the acceptor.^34^ Furthermore, time-dependent DFT (TD-DFT) has been extensively used to calculate the properties of excited states and absorption spectra which have been used to get insights into the light-harvesting ability of NFAs.

**Fig. 3:**
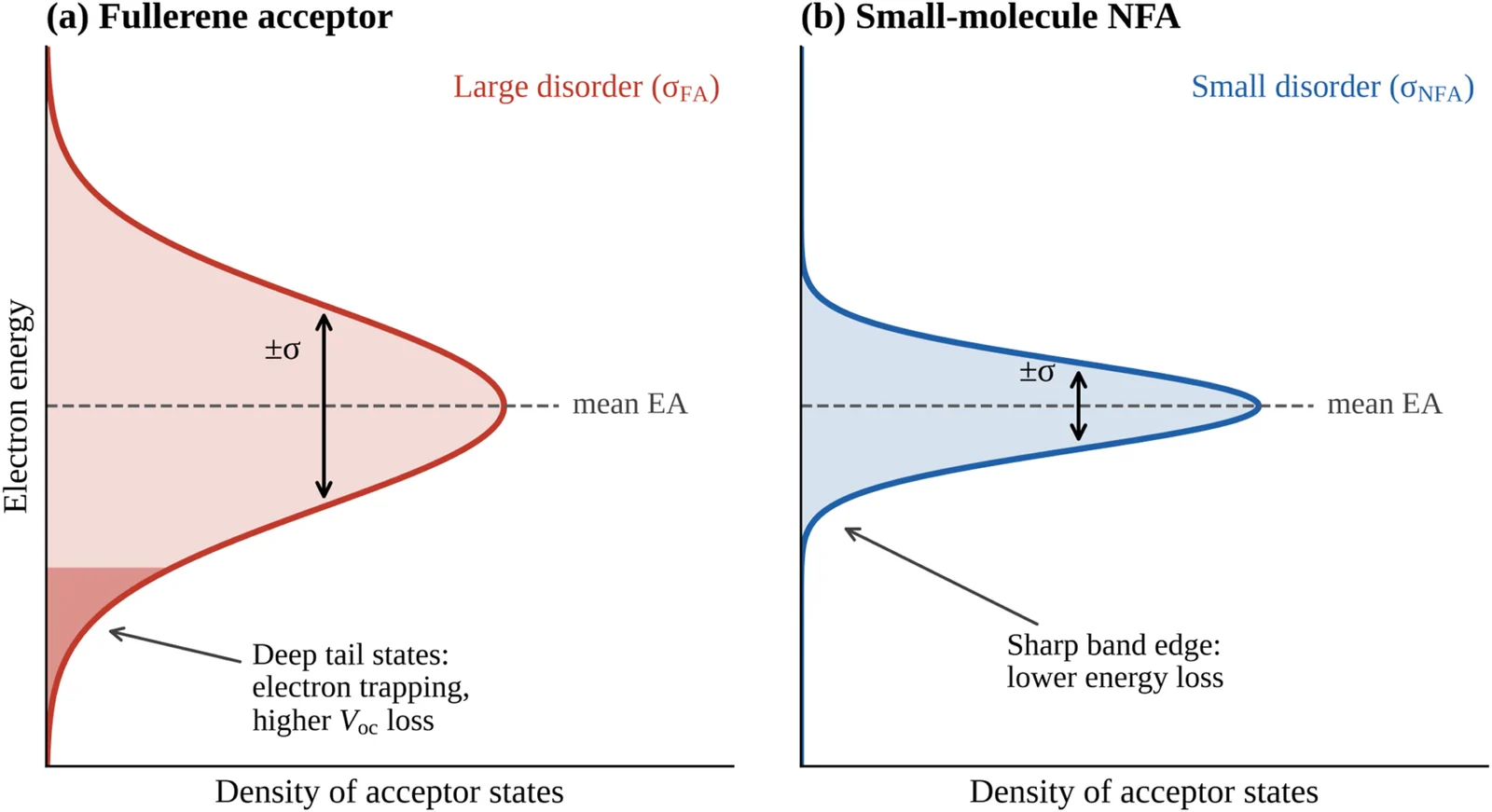
Schematic illustration of the distribution of EA (density of acceptor states) in (a) fullerene acceptors and (b) small-molecule NFAs. The broader distribution in fullerene acceptors reflects a larger energetic disorder (*σ*) and deeper tail states, whereas the small-molecule NFAs show a narrower distribution with a sharper band edge. The schematic is drawn by the authors on the basis of the results reported in ref. 19.

The molecular structure, morphology, intermolecular interactions and molecular packing in the BHJ of OSCs have been extensively explored using MD simulations.^32,35,36^ These simulations provide valuable information on donor–acceptor interactions, charge transport, and device performance while revealing the effects of side-chain engineering and molecular aggregation.^37^ The MD studies also provide information about the reaction kinetics, charge-carrier dynamics, and evolution of morphology in the active layer. Interestingly, even minor differences in the side-chain structure can have a marked effect on the molecular packing, intermolecular interactions, and charge transport and hence on the photovoltaic properties.^30^ Hence, the MD simulations have become a necessary tool for designing high performance NFAs that possess higher charge transport and device efficiency. ML models have been powerful tools to predict the absorption coefficients, energy levels, exciton dissociation and charge transport rates of NFAs. The relevant algorithms and their generative models can be applied to design NFA structures, simulate their performance and identify potential candidates for OSCs.^38^ These features can efficiently utilize the resources and time required for physical screening by bringing a balance between accuracy and computational cost. It has been revealed that the PCE, optical and structural properties of solar cells can be predicted by ML based on the alignment of acceptor and donor units.^39^ The results showed that generative adversarial networks (GANs) are a very efficient method to design NFA structures with the desired properties.

To date, several reviews are available to shed light on the current status of research related to organic solar cells.^40,41^ For instance, the chemistry of non-fullerene acceptors and the design principles of fused-ring electron acceptors,^42,43^ the physical processes related to performance of NFA-based devices^44^ and the stability of organic solar cells^45^ have been comprehensively described. However, the literature survey points to deficiency in application of computational approaches, *i.e.* DFT, MD simulations and ML, to shed light on the degradation pathways of NFAs. The present review is dedicated to fill this gap by highlighting the design strategies, breakthroughs related to utilization of NFAs, challenges and opportunities. The survey of key tools, principles, and techniques employed in designing of NFAs, with insights from DFT calculations, MD simulations and the emerging ML models, is comprehensively described. The analysis of computational approaches being implemented in designing NFAs in order to enhance the PCE of OSCs is specifically given. This review mainly considers small-molecule non-fullerene acceptors (SM-NFAs), which are different from the polymer acceptors employed in all-polymer solar cells, because they have different molecular design principles, synthesis methods and well-defined optoelectronic properties, whereas the polymerized acceptors are briefly discussed in Section 8.4. Finally, to address the paramount challenge of device longevity, Section 9 provides a dedicated analysis of NFA degradation processes (including photo, thermal, morphological, and oxidative pathways). The ways in which computational techniques, ranging from first-principles DFT to MD simulations, identify structural vulnerabilities and drive the design of degradation-resistant acceptor materials are also highlighted.

## 2. Architecture of non-fullerene acceptors (NFAs)

The use of NFAs has revolutionized the field of OPVs, as they are more flexible, cost efficient and have a wider absorbance spectrum than traditional fullerene acceptors. Recently, efforts have been made to achieve high photovoltaic performance with acceptor–donor–acceptor (A–D–A) and D–π–A architectures.^46^ A representative A–D–A acceptor ITIC consists of an electron-rich fused-ring core (indacenodithieno[3,2-*b*]thiophene, IDTT) and two electron-deficient terminal units (2-(3-oxo-2,3-dihydroinden-1-ylidene)malononitrile, INCN), as shown in Fig. 4.^27^ The optimization of the energy levels and absorption properties of these structures increases the push–pull effect and PCE. The electronic and optical properties of NFAs can be tuned by introducing the electron deficient ladder-type cores like quinoxaline, benzotriazole, and benzothiadiazole.^47^ At that stage, the highest PCE values (around 16%) were recorded for the advanced A–DA′D–A type acceptors, namely, Y6, Y5 and BZIC. A similar Y1-based NFA, incorporating a BTA-based acceptor with the donor HFQ_*x*_-T and extending the central core with thieno[3,2-*b*]thiophene, demonstrated a PCE of almost 14%.^36,48^

**Fig. 4:**
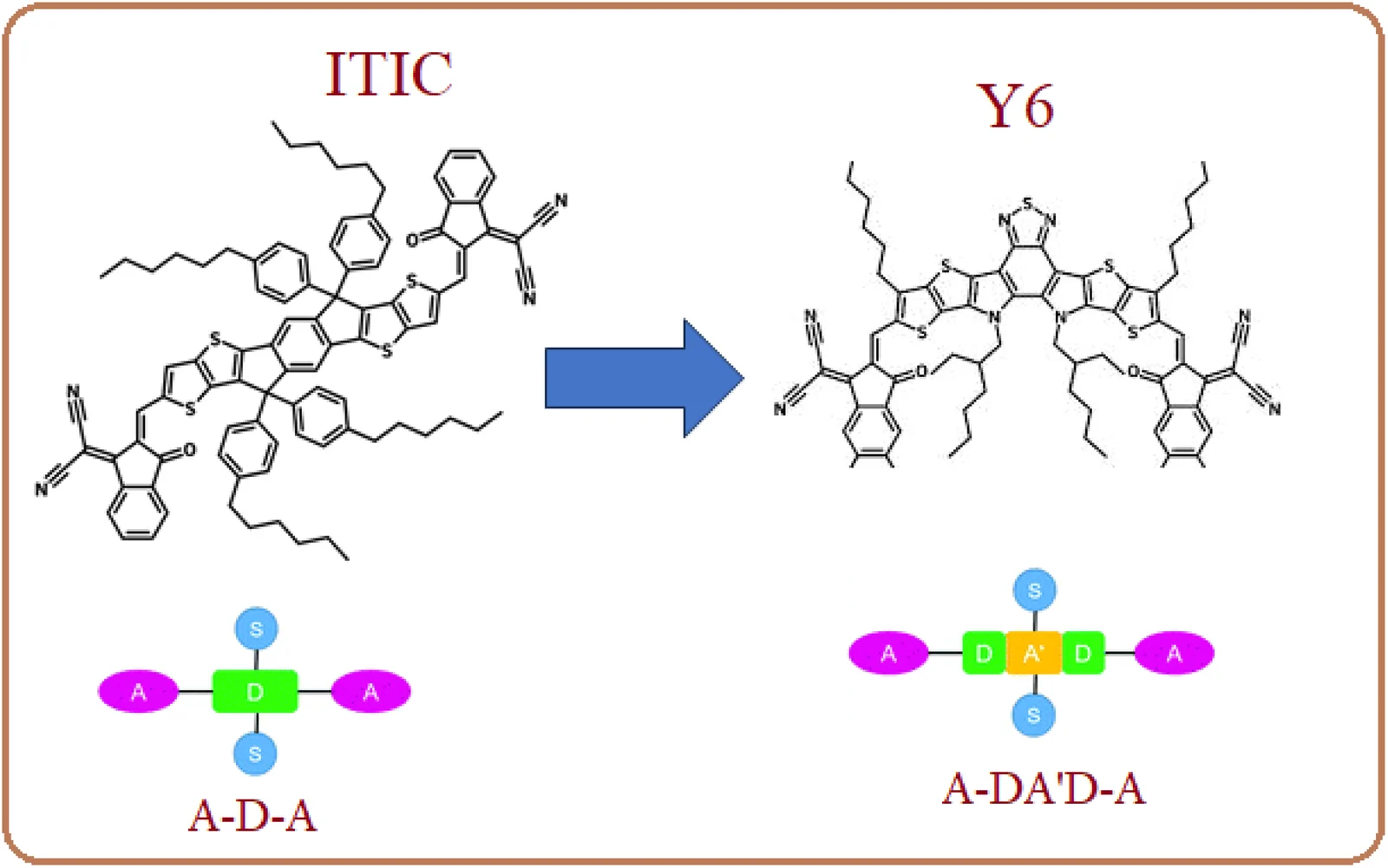
Examples of A–D–A and A–DA′D–A non-fullerene acceptor molecules. This figure has been adapted/reproduced from ref. 49 with permission from Royal Society of Chemistry, copyright 2020.

A number of A–DA′D–A-type NFAs are now available with PCEs in the range of 15% to 17% with appropriate donor materials.^49^ Their electron deficient ladder structures enable effective charge transport and enable this structure to be one of the most successful designs for high performance organic solar cells. In addition to the basic A–D–A and A–DA′D–A architectures, three important molecular engineering strategies have greatly enriched the design of advanced NFAs. The replacement of S with Se increases the polarizability due to the larger atomic radius, which results in a red-shift of the absorption (up to 50 nm bathochromic shift with Se-substituted Y6) and better charge transport, which is facilitated by stronger intermolecular interactions (with Se-substituted Y6 variants showing PCE improvements), as well as higher crystallinity and lower reorganization energy.^35,39,40^ Side-chain engineering, *i.e.* the optimization of the branching position, fluorination pattern and length of alkyl chains, provides precise control of solubility, packing and morphology of molecules which consequently influence molecular orientation, domain purity, crystallinity and device stability.^50,51^

The development of the A–DA′D–A-type NFAs with the evolution of the Y1 and Y6 acceptors is shown in Fig. 5.^52,53^ The accepting units have comparatively large molecular backbones, which restricts the delocalization of electrons. However, by coupling the donor to the acceptor *via* a π-bridge, the limitation is reduced and can be even eliminated, especially by increasing the length of the π-bridge in the connection.^54^ Commonly used donor groups are phenyl and fluorinated groups whereas π-bridges such as alkyl-thiophene, cyclopentadithiophene, benzothiadiazole and thienyl are commonly used.^50,55^ The π-bridge increases the regularity of the molecular plane, enhances π-electron delocalization and regulates molecular charge transport, which finally improves the PCE.^51,56^ Thus, D–π–A type NFAs have a very wide optical absorption, high charge transport efficiency and a good energy level alignment configuration.^57,58^

**Fig. 5:**
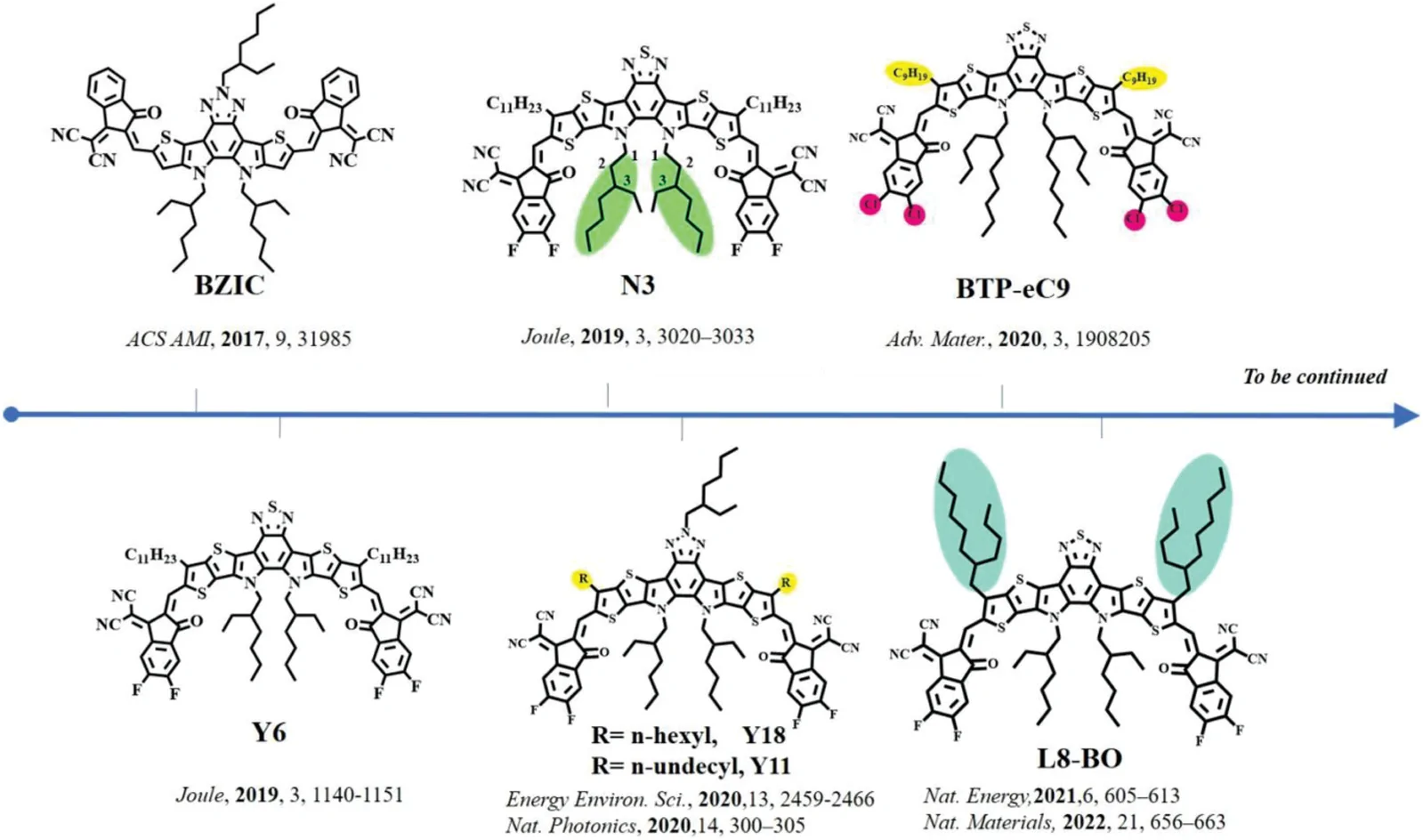
Origin of the A–DA′D–A structure and the synthetic pathways of Y1 and Y6 NFAs. This figure has been adapted/reproduced from ref. 59 with permission from John Wiley and Sons, under creative commons CC BY license 2023.

### 2.1 Energy levels in NFAs

For the efficient charge separation and minimal energy loss in organic solar cells, it is imperative that the energy levels of the two materials be aligned.^60,61^ Proper EA and ionization energy allow for easier exciton dissociation and reduced recombination losses. The losses are primarily due to band gap recombination (0.26–0.30 eV) and sub band gap absorption related to interfacial charge transfer (around 0.2 eV).

Exciton binding energy and driving force for charge generation is related to the EA of donor and acceptor materials.^63^ In optimized donor–acceptor combinations, the energy offsets are minimized while maintaining efficient charge separation. Polymer NFA blends typically show lower longitudinal absorption in the tail state which means that absorption at the interfaces between the polymer and NFA is low and its charge transport is efficient.^64^ Besides, electronic coupling and molecular packing are also important to suppress charge recombination and increase the photovoltaic performance.^65^ As shown in Fig. 6, formation of the interfaces affects the energy levels alignment, lowers the interfacial energy offset, and raises the photovoltaic (PV) gap. For this reason, energy-level alignment and molecular packing designed in a controlled way are crucial to boost both exciton dissociation and charge transport/dissociation as well as to increase the light-absorption within the NFA.^66,67^

**Fig. 6:**
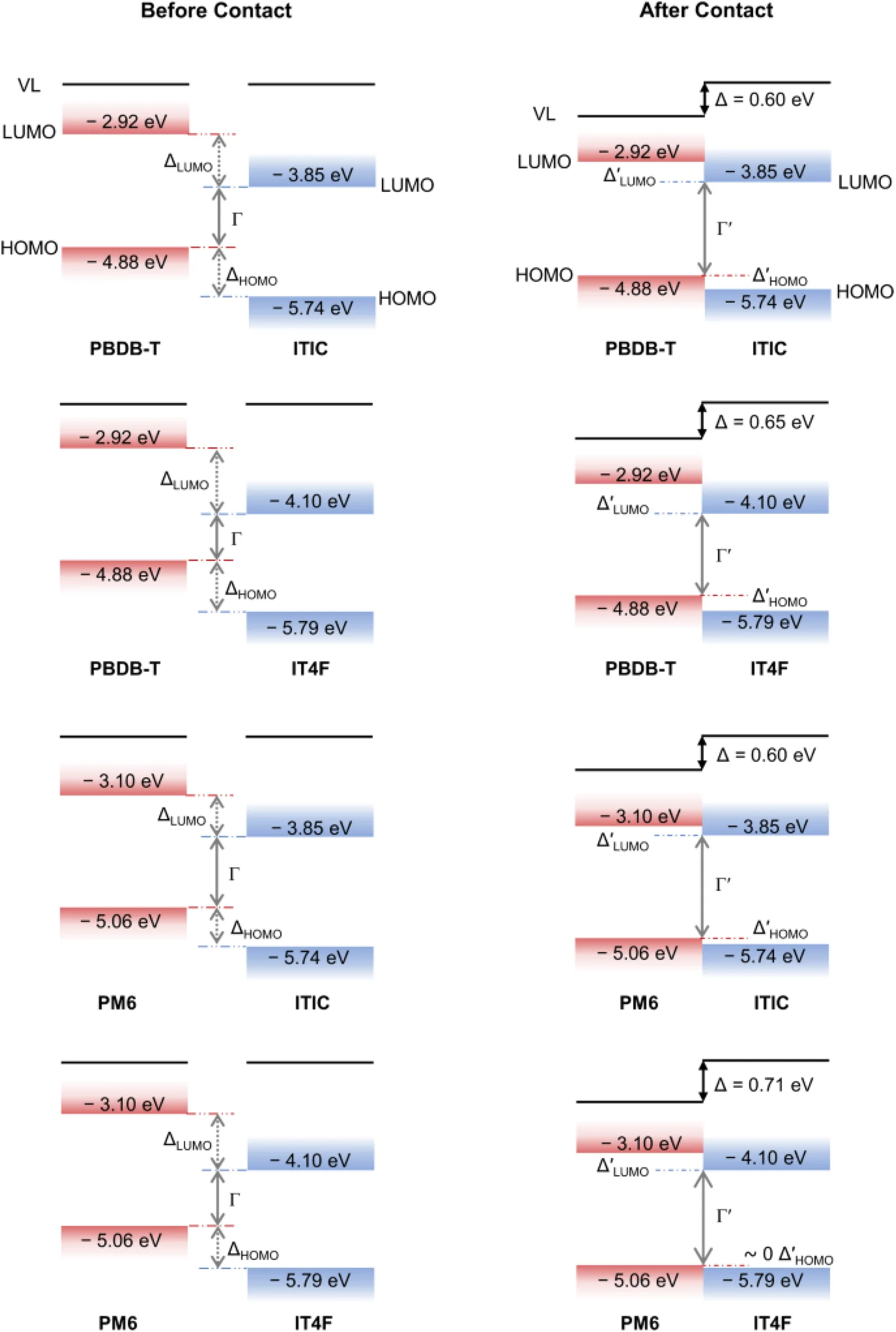
Alignment of the energy levels of donor and acceptor molecules with respect to the vacuum level before and after the formation of the interface, along with the potential charge separation in OPVs. This figure has been adapted/reproduced from ref. 62 with permission from Springer Nature, under creative commons CC BY license 2023.

## 3. Study of OSCs *via* density functional theory

First-principles computational methods, such as DFT, have been widely used to investigate the electronic structure and optical properties of molecular materials, as indicated in Fig. 7.^68^ DFT, which is based on the Hohenberg–Kohn formalism, calculates material properties by minimizing the total energy of the system as a function of the density of the electrons.^69–71^ Due to its accuracy and computational efficiency, DFT has emerged as a valuable computational method to study the chemical and physical properties of bulk and low-dimensional materials.^68^ DFT is widely used in OPV to study NFAs and interaction between NFA and donor materials to rationally design high performance OPV.

**Fig. 7:**
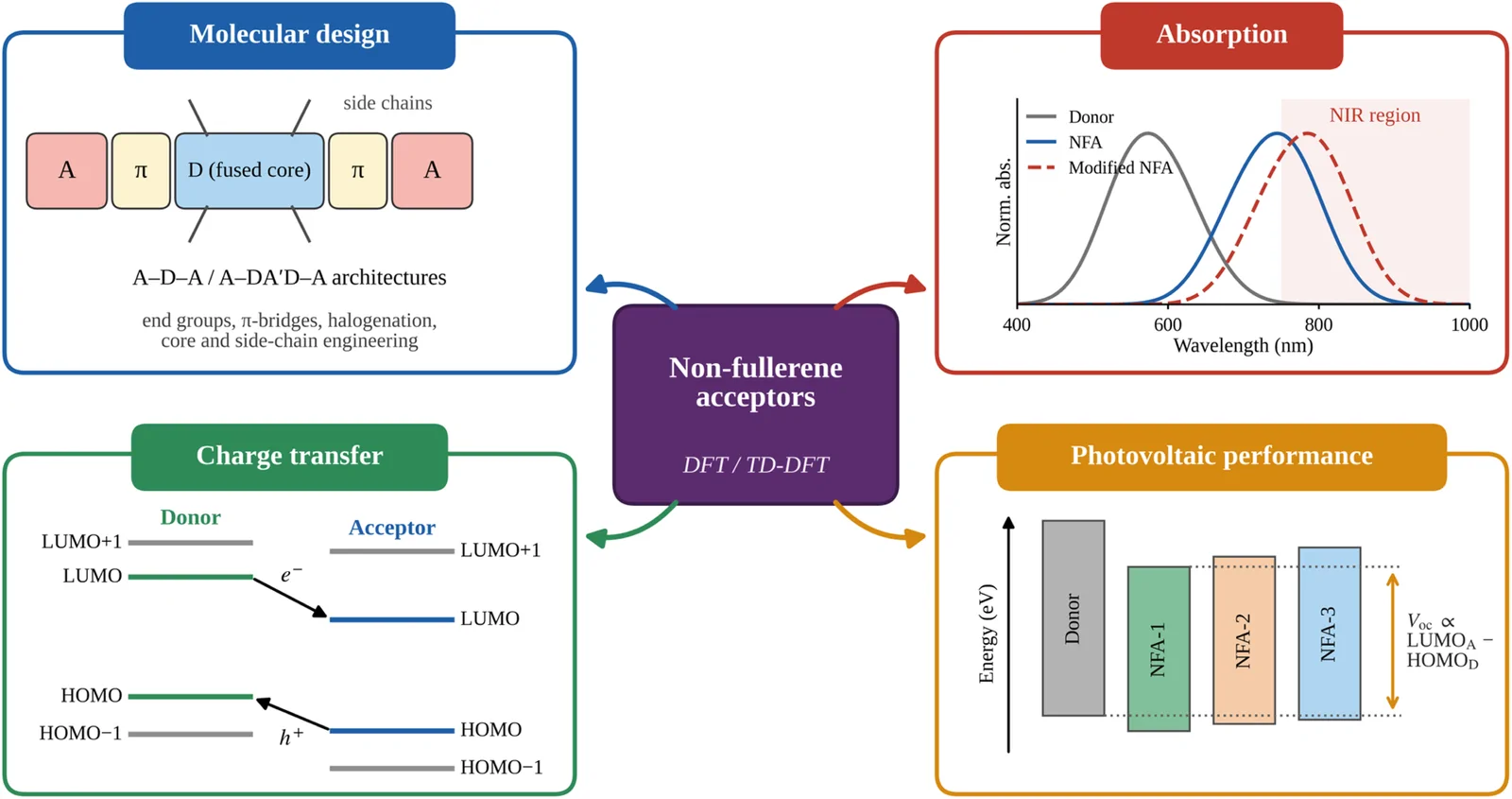
Schematic illustration of the key aspects of NFAs investigated by DFT and TD-DFT calculations, *i.e.* molecular design, absorption, charge transfer and photovoltaic performance.

Before proceeding to further calculations, optimization of the ground state molecular geometry has to be accurate to predict electronic and optical properties. Better accuracy in DFT calculations also crucially depends on the use of an appropriate exchange–correlation (XC) functional, such as conventional local density approximation (LDA) and generalized gradient approximations (GGA) to the functional, or recent hybrid functionals.

### 3.1 Overview of exchange–correlation approximations

The accuracy of DFT calculations depends on the choice of the XC functional. The XC functionals are classified in accordance with the Jacob's ladder which mentions LDA, GGA, meta-GGA, global hybrid and range-separated hybrid (RSH) functionals. The climbing in this ladder does not guarantee a better description of every property which demands care in application of the functionals to different materials. In case of NFAs, the choice of the functional should be made by considering the property of interest and validated against experimental data. In this section, the main classes of functionals along with their appropriate use for NFA systems are briefly introduced, as summarized in Table 1. The critical evaluation of these functionals against experimental benchmarks is given in Section 3.2.

**Table 1:** Characteristics and appropriate use of different classes of exchange–correlation functionals for NFA systems^a^

Functional class	Typical examples	Exact (HF) exchange	Appropriate use for NFAs	Main limitations	Ref.
LDA	SVWN	0%	Not recommended for the electronic structure of NFAs	SIE; severe gap underestimation; no dispersion	72 and 73
GGA	PBE, BLYP	0%	Preliminary geometry or periodic packing, only with dispersion correction (*e.g.* PBE-D3)	Gap underestimated by >1 eV; spurious low-lying CT states	74, 76 and 78
Meta-GGA	TPSS, M06-L	0%	Limited; not reliable for CT properties	Residual SIE	78
Global hybrid	B3LYP, PBE0	20–25% (fixed)	Geometries, reorganization energies, trends in HOMO/LUMO, localized excitations	Underestimated fundamental gap; incorrect long-range behaviour; poor CT energies	81–83
RSH (default *ω*)	CAM-B3LYP, ωB97X-D, LC-ωHPBE	19–100% (distance dependent)	CT excitations; hole–electron separation analysis	Default *ω* too large for extended NFAs; overestimated gaps	84 and 85
Optimally tuned/screened RSH	Tuned ωB97XD, OT-SRSH-PCM	*ω* tuned; long range screened by dielectric constant	IP, EA, fundamental gap and CT energies; solid-state gaps	Tuning required for each molecule	87–89

a CT: charge transfer; HF: Hartree–Fock; IP: ionization potential; EA: electron affinity; RSH: range-separated hybrid; SIE: self-interaction error; OT-SRSH-PCM: optimally tuned screened RSH with polarizable continuum model.

#### 3.1.1 Semi-local functionals (LDA, GGA and meta-GGA)

LDA is the simplest approximation, in which the XC energy at each point depends only on the local electron density, taken from the homogeneous electron gas.^72,73^ The limitations of LDA are improved in GGA by including the gradient of the electron density, ∇*ρ*(*r*), whereas *meta*-GGA further includes the kinetic energy density.^72,74,75^ These semi-local functionals are computationally efficient and appeared to give reasonable bond lengths and lattice parameters for several inorganic solids. However, they are not suitable for predicting the electronic structure of large, highly polarizable, π-conjugated NFA molecules. The incomplete cancellation of the self-interaction error (SIE) causes an artificial over-delocalization of electron density along the extended A–D–A and A–DA′D–A backbones. Consequently, the fundamental gap is severely underestimated, often by more than 1 eV (ref. 76) and TD-DFT calculations with these functionals predict spurious low-lying charge-transfer (CT) states. Furthermore, LDA and standard GGAs cannot describe the long-range dispersion interactions which govern the π–π stacking and packing of NFAs.^73,77^ Therefore, semi-local functionals are not suitable for predicting frontier orbital energies, energy gaps, excited states or CT properties of NFAs. These are useful for making preliminary geometry optimization or periodic calculations of molecular packing, and even then only with explicit dispersion corrections like PBE-D3.^78^ After finding optimized geometries, the accurate description of electronic properties needs a higher level of theory.

#### 3.1.2 Global hybrid functionals

Global hybrid functionals partially correct the SIE by mixing a fixed fraction of exact Hartree–Fock (HF) exchange with DFT exchange.^79^ For instance, 20% mixing in B3LYP and 25% mixing in PBE0 may be found helpful. The functional B3LYP is the most widely used functional in the NFA literature as it gives reliable ground-state geometries, reorganization energies, and trends in HOMO/LUMO energies within a series of structurally related molecules.^80,81^ In case of localized π → π* excitations, B3LYP often reproduces the experimental absorption maximum reliably. For example, among four functionals applied to an asymmetric NFA, the *λ*_max_ value predicted by B3LYP (682 nm) has been found closest to the experimental value as indicated in Fig. 8.^82^ However, such agreement partly results from a cancellation of errors, and it should not be generalized. Since the fraction of HF exchange is constant at all inter-electronic distances, global hybrids do not recover the correct −1/*r* asymptotic behaviour of the exchange potential. Hence, they still underestimate the fundamental gap and the energies of long-range CT excitations, and their performance depends strongly on the fraction of HF exchange.^83^ B3LYP appeared inaccurate in describing dispersion interactions without employing the empirical corrections.

**Fig. 8:**
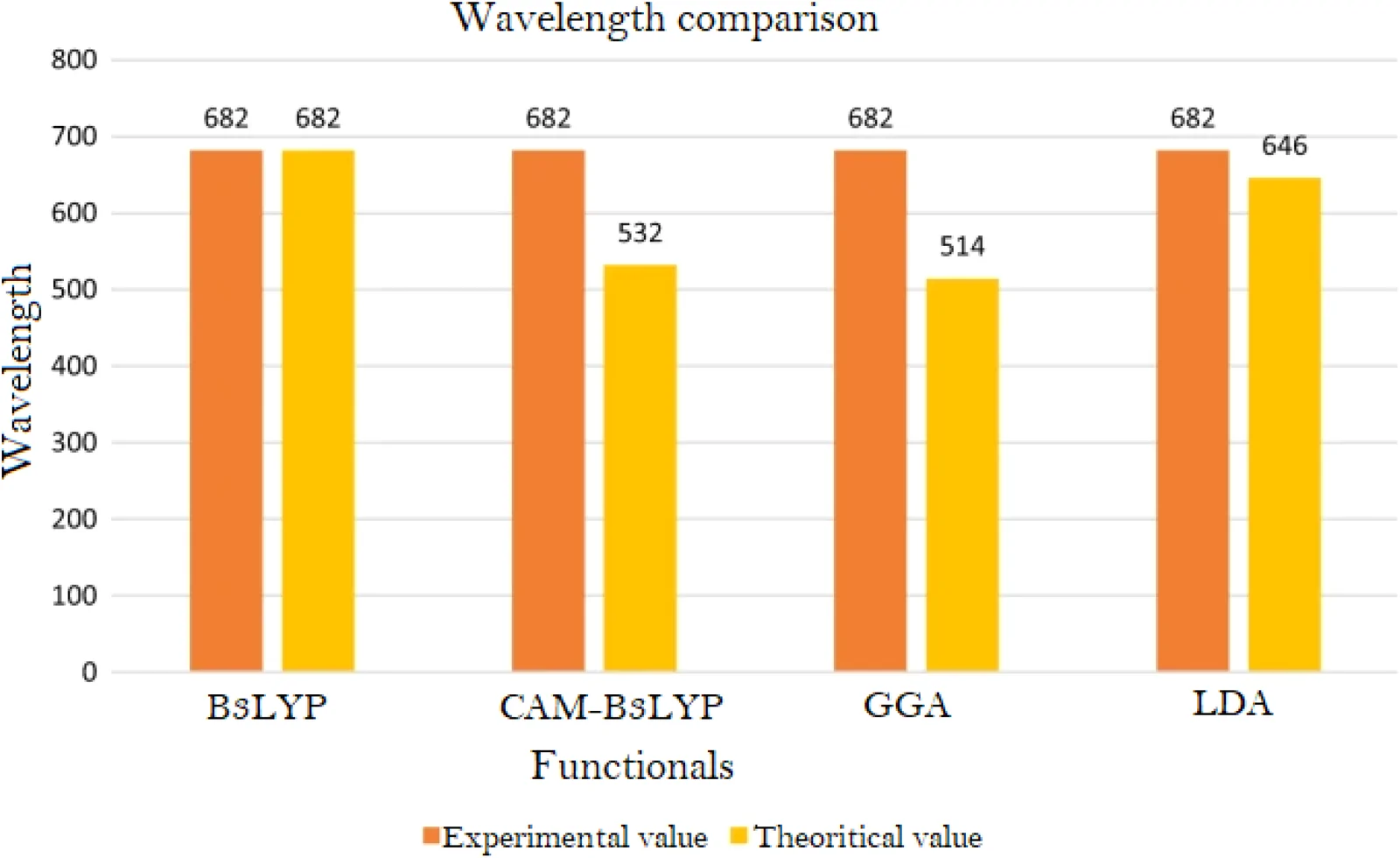
Comparison of the experimental and theoretically calculated absorption wavelengths of an asymmetric NFA obtained using different DFT functionals.^82^

#### 3.1.3 Range-separated hybrid functionals and optimal tuning

RSH functionals have been developed to address the issue of incorrect long-range behaviour of global hybrids. In RSH functionals, the Coulomb operator is partitioned into short-range and long-range parts through the error function, with the range-separation parameter *ω* controlling the transition between them as given in eqn (1) in Section 3.2.2. The short-range exchange is treated mainly by DFT, whereas the long-range exchange is treated increasingly by HF exchange, so that the correct asymptotic behaviour is recovered. In this regard, the examples are CAM-B3LYP, which contains 19% HF exchange at short range and 65% at long range, and ωB97X-D and LC-ωHPBE, which reach 100% HF exchange at long range. These functionals give a much better description of CT excitations and of the spatial separation of hole and electron densities in NFAs, and they are therefore preferred for excited-state and CT analysis.^84,85^ A variation of this approach appeared in the form of local RSH which employs a position-dependent screening parameter to improve the short-range description.^86^

The default *ω* values are optimized for small molecules, and they are often too large for extended and highly polarizable NFAs. Consequently, RSH functionals with default *ω* value tend to overestimate the gaps and blue-shift the absorption of these molecules. This issue is handled by optimal tuning in which *ω* is determined non-empirically for each molecule so that the HOMO energy equals the negative of the ionization potential. A functional ωB97XD tuned in this way has been used to study A–D–A′–D–A type NFAs, and the tuned functional predicted *λ*_max_, exciton binding energy, ionization potential (IP), EA and reorganization energy with small deviations from experiment.^87^ The dependence of the results on the fraction of HF exchange has also been examined systematically. In a TD-DFT study of a series of NFAs using several functionals, the functionals containing about 20% HF exchange performed similarly to B3LYP, whereas the functionals with a higher fraction of HF exchange exhibited notable deviations.^83^

A comprehensive benchmark of 55 functionals has been reported for the A–D–A acceptor ITIC and the A–DA′D–A acceptor Y5 (Fig. 9), considering their optical gaps and reduction and oxidation potentials.^78^ It was observed that no single functional described all the properties best. The functionals tHCTHhyb, M11L and OHSE2PBE predicted both redox potentials of ITIC with errors between −0.22 and 0.05 eV, and these functionals performed well for Y5 too. The same study showed that the gas-phase fundamental gap given by an RSH functional must be corrected for the polarization of the surrounding medium before it is compared with the solid-state experimental data. This is achieved by the screened RSH approach discussed in Section 3.2.3.

**Fig. 9:**
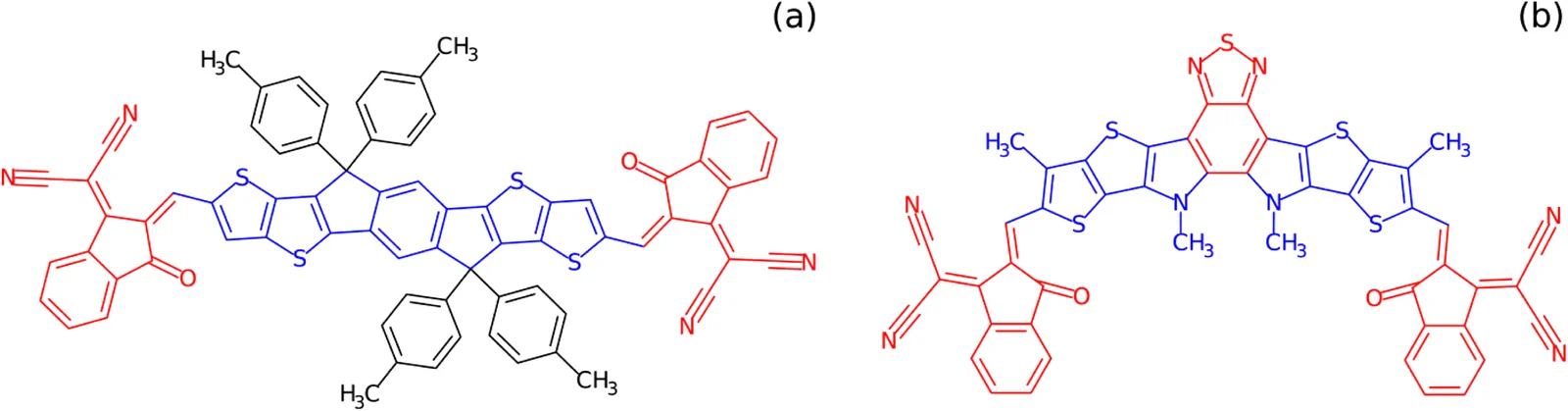
Chemical structures of the NFAs (a) ITIC and (b) Y5, in which the alkyl chains are substituted with methyl groups. The intramolecular acceptor (A) units are shown in red, whereas the donor (D) units are shown in blue. This figure has been adapted/reproduced from ref. 78 with permission from AIP Publishing under creative commons CC BY license 2023.

Table 2 summarizes the experimentally reported photovoltaic parameters of representative donor and acceptor materials along with the functionals used in their computational investigation. When blended with appropriate donor polymers, the Y-series acceptors, especially Y6 and its derivatives, have proven to be the most successful class of NFAs with PCE values over 15%.^90^ The polymer donor D18, when blended with Y-series acceptors such as L8-BO, appeared to yield PCE close to 19.5% with low energy loss. The DFT calculations have been found helpful in explaining the favourable energy level offset in these blends.^91^ More recently, the *o*-BTP-eC9 and asymmetric side-chain acceptors have emerged as the next generation of NFAs, where DFT and MD simulations facilitated the optimization of molecular packing and the reduction of energy loss.^92,93^

**Table 2:** Experimentally reported photovoltaic parameters of representative donor and non-fullerene acceptor materials along with the functionals used in their computational investigation^a^

Component category	Comp. name	Functionals used	Key properties	PCE (%)	FF (%)	*J*_SC_ (mA cm^−2^)	Ref.
Polymer donors	PM6	B3LYP	High hole mobility, large absorption spectrum	15.5	78	21.5	94
	D18	PBE0	High hole mobility, high crystalline stability	17.3	81	23.3	95
Experimental non-fullerene	IDIC	B3LYP	High electron mobility, narrow band gap	6.5	72	14.2	96
	ITIC	PBE0	Strong absorption in the visible range, high electron affinity	6.8	74	15.1	97
	Y6	PBE0	Strong absorption, low band gap (1.3 eV), excellent stability	15.2	80	22.1	90
Acceptors (NFAs)	*o*-BTP-eC9	B3LYP, CAM-B3LYP	Energy loss reduction (41 meV); enhanced stability	19.9	79	26.5	92
	Asymmetric side-chain acceptor	DFT + MD	Optimized packing; π–π: 3.52 Å; mobility: 3.8 × 10^−4^	20.1	80	26.8	93
	D18:L8-BO	PBE0, B3LYP	*E*_loss_ < 0.5 eV; optimal energy offset (0.97 eV)	19.5	78	26.2	91

a All PCE, FF and *J*_SC_ values given in table are experimentally reported device parameters taken from the cited literature. Besides polymer donor:NFA blends, the non-fullerene acceptors have also been utilized in all-small-molecule OSCs.^98^ The perylene diimide based polymer acceptor reported in 2007 was the first polymer acceptor used in all-polymer solar cells,^25^ whereas the concept of fused-ring electron acceptors was introduced with the planar acceptor IDIC which delivered 8.71% efficiency in as-cast devices.^26^ These developments laid the foundation of next-generation OPVs based on NFAs.^24^ Moreover, computational molecular engineering has been used to design new donor molecules,^99^ and manipulation of molecular stacking to prolong the exciton diffusion length has helped to achieve efficiency over 19% in pseudo-planar heterojunction devices.^100^ The chemistry of NFAs and the design principles of fused-ring electron acceptors have been reviewed comprehensively,^42,43^ while the physical processes behind the performance of NFA based devices have been discussed in detail.^44^ It has been observed that the intramolecular charge-transfer character of excitons influences the polaron generation at the donor/acceptor interface,^101^ and enhanced exciton dissociation has even allowed efficient single-material solar cells.^102^ The design rules for molecular donors have also been established^103^ and the challenges related to the stability of OSCs along with strategies to overcome them have been reviewed.^45^ Furthermore, the Voc can be tuned by desymmetrization of A–D–A molecules^104^ and the voltage loss can be reduced by increasing the donor–acceptor spacing.^105^ The A–D–A architecture has also been exploited in vacuum-processed molecular donors,^106^ whereas the emergence of Y-series acceptors has opened a new phase in OSC research.^107^

### 3.2 Critical selection of density functionals for NFA architectures

The accurate modeling of non-fullerene acceptors (NFAs), particularly the widely studied A–D–A and A–DA′D–A type architectures (*e.g.* ITIC and Y6), presents an exceptional challenge to conventional electronic structure methods.^108–110^ Because these systems are characterized by extended, highly polarizable π-conjugated frameworks, intricate π–π stacking and strong intrinsic CT characteristics between the core and endcap units, the choice of the exchange–correlation functional is not merely a matter of computational convenience.^111^ Rather, it fundamentally governs the validity of the predicted physical properties. Treating density functionals as a generic “menu” of exchange–correlation approximations frequently leads to severe qualitative and quantitative errors in the NFA literature.

#### 3.2.1 Breakdown of semi-local functionals (LDA and GGAs)

Standard semi-local approximations, encompassing the LDA and GGAs such as PBE and BLYP, are fundamentally inadequate for the predictive modeling of NFA systems. Their failure stems from two deep-seated theoretical deficiencies. The first one is the inability to describe long-range dispersion. NFAs rely heavily on non-covalent, non-local van der Waals (vdW) interactions to dictate their solid-state film morphology, packing orientations, and intermolecular transfer integrals. Because semi-local functionals only utilize local charge densities and their gradients, they completely fail to capture the long-range electron correlation responsible for dispersion. Consequently, uncorrected GGAs yield unphysically elongated intermolecular distances and severe underestimations of binding energies in NFA dimers and crystals. To obtain meaningful morphological structures, the explicit inclusion of empirical or semi-empirical dispersion corrections (such as Grimme's DFT-D3 (BJ) or DFT-D4, or non-local vdW functionals like vdW-DF2) is necessary.^112,113^

The second one is the SIE and the related delocalization artefacts. Semi-local functionals suffer from severe SIE due to incomplete cancellation of the artificial electron self-repulsion. In the extended π-systems of NFAs, SIE manifests as a fictitious over-delocalization of electron density. This artificially stabilizes the frontier molecular orbitals, leading to a drastic underestimation of the fundamental electronic gap often by 1.0 eV or more compared to gas-phase experimental benchmarks.^114^ Crucially, this delocalization error undermines TD-DFT simulations of excited states. Because standard GGAs cannot capture the correct long-range asymptotic behavior (−1/*r*) of the exchange potential, they catastrophically underestimate the energy of charge-transfer excited states, frequently yielding spurious, non-physical low-lying CT transitions that skew the predicted absorption spectra.^115^

#### 3.2.2 Global hybrids and the necessity of range separation

Global hybrid functionals, such as B3LYP (containing 20% exact HF exchange) or PBE0 (25% HF exchange), partially mitigate the SIE by incorporating a fixed fraction of exact exchange. This inclusion contracts the artificially delocalized wavefunctions and improves the prediction of fundamental gaps and locally excited (LE) valence transitions. However, global hybrids remain structurally limited when applied to larger NFA molecules. Because the fraction of HF exchange is constant across all inter-electronic distances, these functionals still exhibit incorrect asymptotic decay at long range. For an extended A–D–A molecule where an electron is transferred across the conjugated core to the endcap, global hybrids undercompensate for the long-range potential, failing to quantitatively capture the binding energies of these spatially separated electron–hole pairs.^108,112,116,117^

To resolve this bottleneck, RSH functionals (*e.g.*, ωB97X, LC-ωHPBE, CAM-B3LYP) have emerged as the modern computational standard for NFA systems. RSH functionals partition the Coulomb operator into short-range (SR) and long-range (LR) components using a screening parameter (*ω*), as expressed by the standard error function relation in eqn (1):^118^1
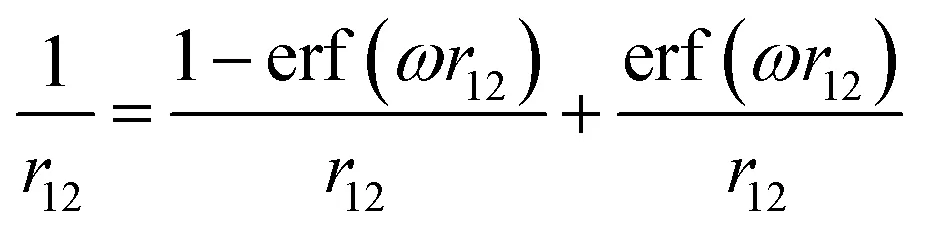
In this framework, the short-range component is treated *via* standard DFT exchange, while the long-range component transitions to 100% Hartree–Fock exchange (*α* + *β* = 1). This mathematically restores the exact −1/*r* asymptotic behavior at long range, which is absolutely vital for describing the electrostatic attraction of a highly extended CT state.^119^ Crucially, the default, pre-parameterized values of the screening parameter (*ω*) are often optimized for small organic molecules and may not fit the highly polarizable, extended structures of modern NFAs. Therefore, implementing first-principles optimal tuning of *ω* is required. This system-specific tuning adjusts *ω* non-empirically for a given NFA geometry until it satisfies the IP theorem (Koopmans' theorem in DFT) given in eqn (2):^120^

2 *ε*_HOMO_ (*ω*) = −IP(*ω*) = −[*E*(*N* − 1) − *E*(*N*)]

By enforcing the condition of eqn (2) for both the neutral (*N*) and anionic (*N* + 1) states simultaneously, the SIE is minimized dynamically for the specific molecular environment. Optimally tuned RSH functionals have been shown to deliver quantitative accuracy, reducing errors in the fundamental gap and orbital energy levels to within 0.1–0.2 eV of high-level GW many-body perturbation theory and experimental measurements.^121^

#### 3.2.3 Screened RSH functionals and solid-state gap renormalization

The optimally tuned RSH functionals discussed above describe an isolated molecule in the gas phase. In a thin film, however, the electronic polarization of the surrounding molecules stabilizes the added hole and electron, which reduces the fundamental gap as compared to its gas-phase value. This effect is known as gap renormalization.^88^ It is taken into account in the screened RSH (SRSH) approach, in which the exact exchange is described by three parameters: *α* is the fraction of exact exchange at short range, *α* + *β* is the fraction at long range, and *ω* controls the transition between the two. In SRSH, *α* + *β* is set equal to 1/*ε*, where *ε* is the dielectric constant of the medium, so that the long-range potential decays as −1/(*εr*), as expected for a screened Coulomb interaction.^88,89^ When combined with a polarizable continuum model (PCM), this approach yields the fundamental gaps of condensed-phase organic semiconductors from single-molecule calculations.^89^

The performance of this approach for Y5 and ITIC is compared in Fig. 10. With the default parameters of the LC-ωHPBE functional (*ω* = 0.4, *α* = 0, *β* = 1; DEF-PCM), the gaps of Y5 and ITIC were overestimated by 2.15 and 1.29 eV, respectively. In contrast, the optimally tuned SRSH-PCM approach predicted IP/EA values of −5.64/−3.56 eV for Y5 and −5.72/−3.58 eV for ITIC, in good agreement with the experimental values, although the calculated EA values were still slightly more negative than the experimental ones.^78^ These results show that both optimal tuning and dielectric screening should be included when the calculated energy levels are compared with the measured energy levels of NFA films.

**Fig. 10:**
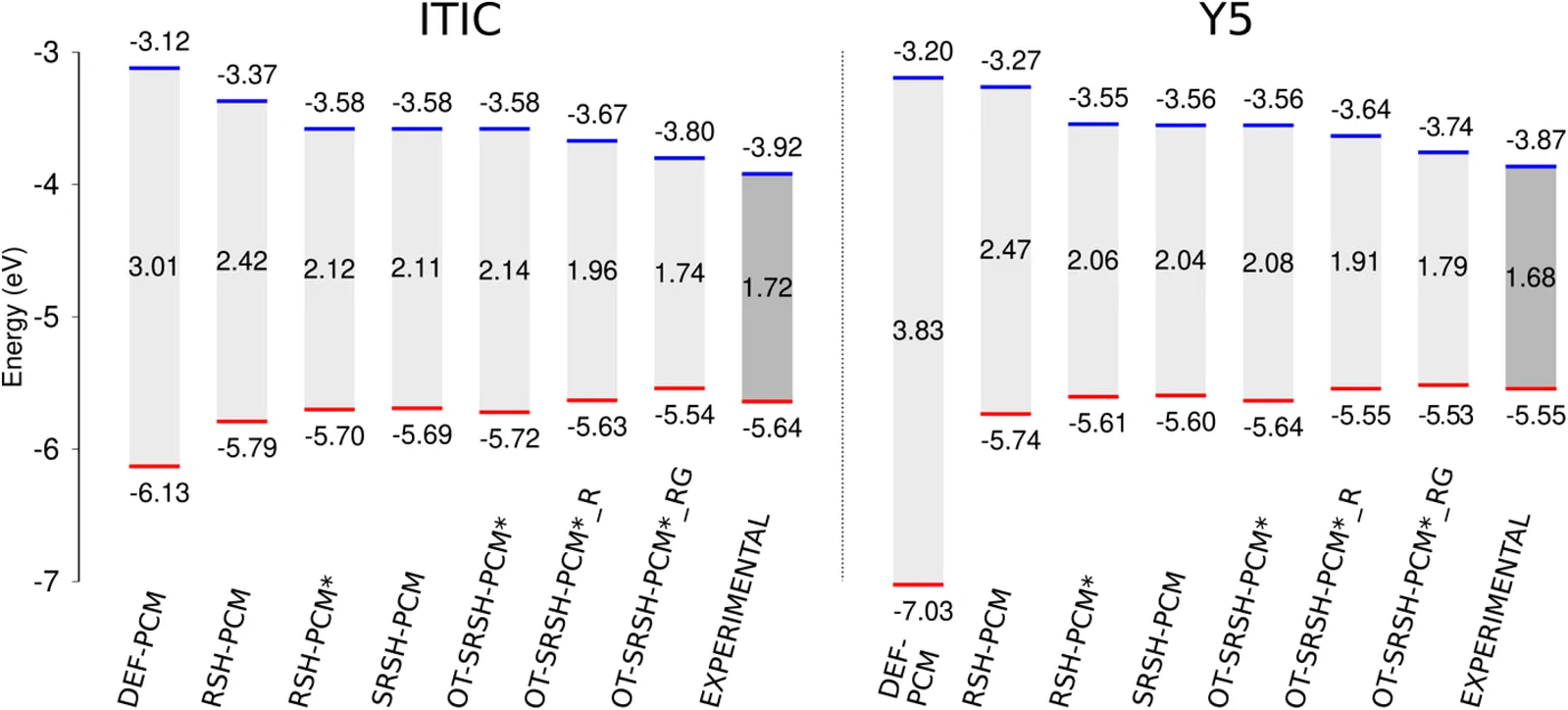
Energy levels of Y5 and ITIC obtained from the RSH and SRSH approaches. DEF represents the LC-ωHPBE functional with default parameters. The experimental reduction and oxidation potentials are given for comparison. This figure has been adapted/reproduced from ref. 78 with permission from AIP Publishing under creative commons CC BY license 2023.

#### 3.2.4 Evaluation against experimental benchmarks

When subjected to rigorous validation against experimental benchmarks—such as gas-phase photoelectron spectroscopy (PES), inverse photoelectron spectroscopy (IPES), electrochemical cyclic voltammetry, and optical absorption profiles, the functional performance hierarchy is clear.

As synthesized from the literature in Table 3, uncorrected semi-local GGAs (PBE) exhibit systematic failures, which severely compress the fundamental gap (*E*_g_) and shift the absorption maximum (*λ*_max_) deep into the infrared due to spurious CT state stabilization. Global hybrids like B3LYP improve the description but still systematically underestimate the fundamental gap by roughly 0.5 eV for flagship acceptors like ITIC and Y6, while overestimating the optical absorption onset due to a lack of proper long-range exchange. Conversely, when the range-separation parameter *ω* is optimally tuned, the resulting RSH calculations excellently match experimental gas-phase IPs and EAs.^122^ Furthermore, TD-DFT calculations performed with tuned RSH functionals successfully reproduce both the precise energetic positioning of the S_1_ vibronic peaks and the mixed local/charge-transfer character of the primary optical transitions without generating artificial electronic states.

**Table 3:** Performance hierarchy of common density functionals against experimental benchmarks for flagship NFA systems

Functional class	Typical examples	Description of vdW interactions	Fundamental gap (*E*_g_) accuracy	TD-DFT excitation/CT description	Primary failure mode/success factor	Ref.
GGA/semi-local	PBE, BLYP	Completely missing (requires explicit D3/D4 corrections)	Severe underestimation (Δ*E* > 1.0 eV)	Catastrophic failure; predicts spurious low-energy CT states	Unphysical electron delocalization due to severe SIE	78 and 123
Global hybrid	B3LYP, PBE0	Missing or severely underestimate	Moderate underestimation (Δ*E* ≈ 0.4–0.6 eV)	Qualitative agreement for localized states; fails for long-range CT	Incorrect asymptotic potential at long ranges; overestimates *λ*_max_	78 and 111
Standard RSH	CAM-B3LYP, ωB97X-D	Often relies on fixed empirical parameters	Variable; depends on molecular size and fixed *ω* value	Improved over global hybrids; can still over/under-shift highly extended CT states	Fixed range-separation parameters fail to adapt to varying NFA polarizability	108
Optimally tuned RSH	Tuned LC-ωHPBE, tuned ωB97X	Highly accurate when combined with modern vdW schemes	High accuracy (Δ*E* < 0.2 eV) *vs.* gas-phase PES/IPES	—	—	89 and 124

## 4. Ground state of NFAs using DFT

The ground-state properties of the NFAs can be well described by DFT (molecular geometry optimization, charge density distribution).^111^ First, DFT can be used to accurately calculate conformation parameters such as bond lengths, bond angles, and dihedral angles of the stable conformations. Subphthalocyanine studies have shown good agreement between experiment and the geometry, charge flow, and device *V*_oc_, obtained at the B3LYP/6-311G(d,p) level.^125^ In addition, it has been demonstrated that the molecules designed with the help of B3LYP calculations can be very efficient for OSC applications.^112^ The stable geometry of different NFA structures can be determined reliably by choosing an appropriate computational recipe.^126^ Furthermore, computational methods can help identify intramolecular interactions, including hydrogen bonding and π–π stacking that can substantially affect the optical and electronic properties of these materials.^127^ This approach has been successfully used in the intermolecular and conformational analysis of highly efficient NFAs such as ITIC.^127^

In terms of the electronic structure, understanding of the mapping of HOMO and LUMO energy levels is critical to understand the charge transfer and *V*_oc_ in the devices. Indeed, investigations of indacenodithiophene-core NFAs have revealed that energy lowering by the incorporation of electron withdrawing units leads to a shift of light absorption to the visible spectral range. In addition, polarizability reflects how easily the electron cloud of an NFA can be distorted in the presence of an external field. In this regard, it has been demonstrated that substitution with electron withdrawing groups (EWGs) on both the donor and the acceptor has the beneficial effect of increasing the polarizability of the NFA molecules. Indenoindene based acceptors such as ZITI and NITI show a PCE of 13% but the strategic use of heteroatoms enables control of energy levels and additional efficiency improvement.^115^ DFT analysis has consistently shown that such structural modifications reduce the band gap and improve the overall efficiency of OSCs, as shown in Fig. 11.^128^ With NFA molecules being so large and complex, it is not always easy to understand their electronic properties. Electronic coarse-graining provides a simplified description of the electronic properties of complex conjugated molecules from their electron density distribution, which is used to predict the electronic properties of the molecules. For instance, this approach has been applied to the A–D–A type NFA ITIC-4F.^112^

**Fig. 11:**
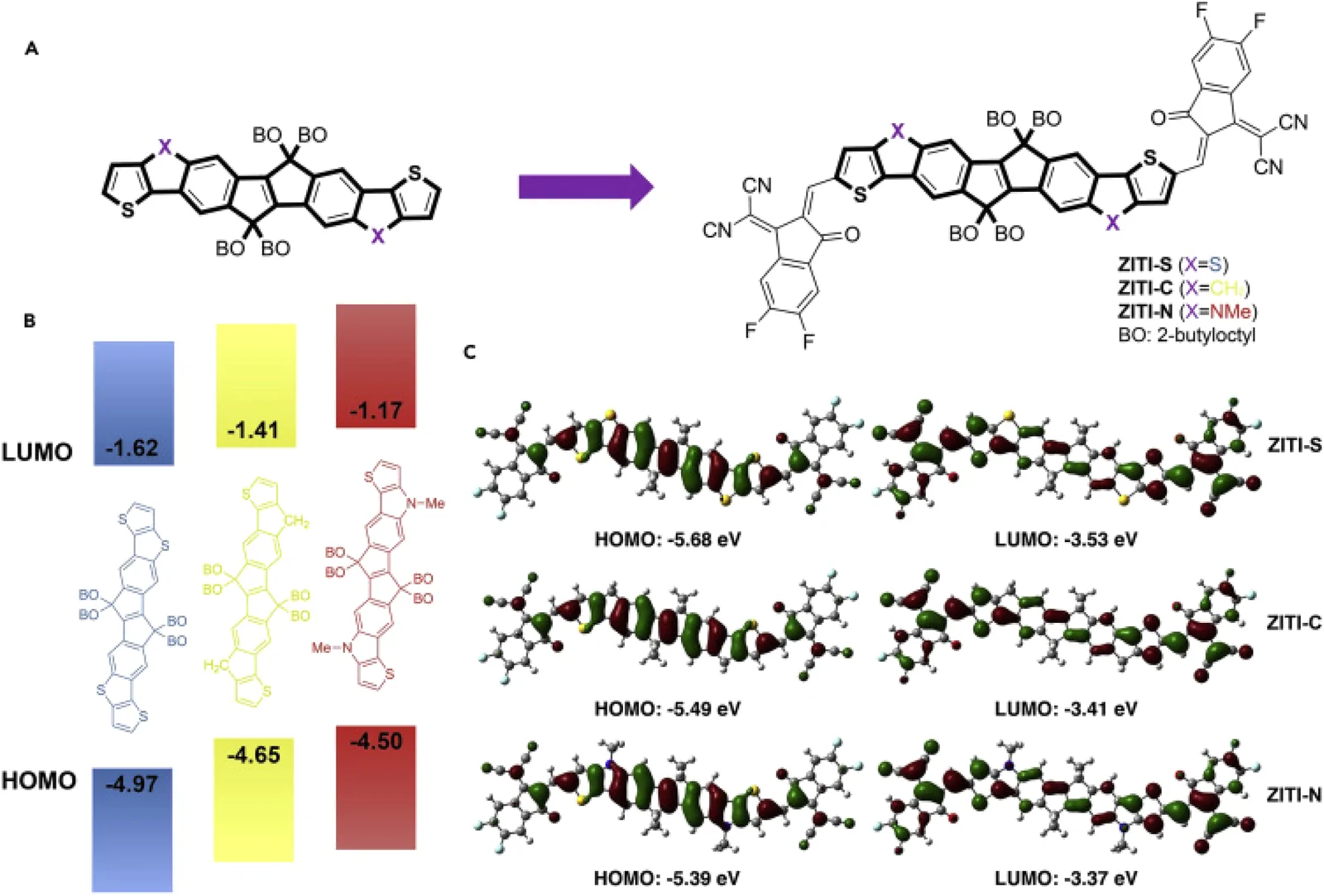
Molecular structure, energy gap and frontier molecular orbitals (FMOs) of the ZITIC molecule. (A) The molecular structures of IIDT-X and ZITI-X. (B) Energy-level evolution of heteroarenes IIDT-S, IIDT-C, and IIDT-N by DFT calculations at B3LYP/6-31G** level. (C) Optimized geometries and contour plots of frontier molecular orbitals with HOMO and LUMO energy levels of ZITI-X NFAs. This figure has been adapted/reproduced from ref. 128 with permission from Elsevier, copyright 2023.

The mapping of the electron density shows that the HOMO is concentrated on the donor unit whereas the LUMO is nearly empty and lies 0.2 eV below the HOMO.^129^ In Fig. 12, the block-wise distribution of the lowest occupied orbitals is shown, where at least two localized molecular orbitals (LMOs) are located at the acceptor unit. The transfer integral between these LMOs is about 120 meV, which is an intermediate value between that of the acceptor–acceptor intermolecular coupling found in materials like IDTBR, ITIC and Y6. If two or more donor LMOs exist, then several pathways for electron transfer are possible because the electron transfer is mediated by a donor unit. To go into these electronic states in more detail, a 6-LMO coarse-grained model, different from the LUMO of the sigma-symmetry subsystem, has been used for visualization of the 2-, 4-, and 6-LMO configurations and their tight-binding (TB) Hamiltonians.^112^ Electronic modeling is an important part of understanding the behaviour of semiconductors in the context of organic solar cells. The electron transport in complex molecular systems is often not properly described by the conventional methods, resulting in uncertainties in the prediction of charge-carrier mobility.^113^ In contrast, coarse-grained modeling can adequately describe the localization of electrons and the flexibility of molecules to accurately analyze charge transport and electronic structures for the design and optimization of NFAs.

**Fig. 12:**
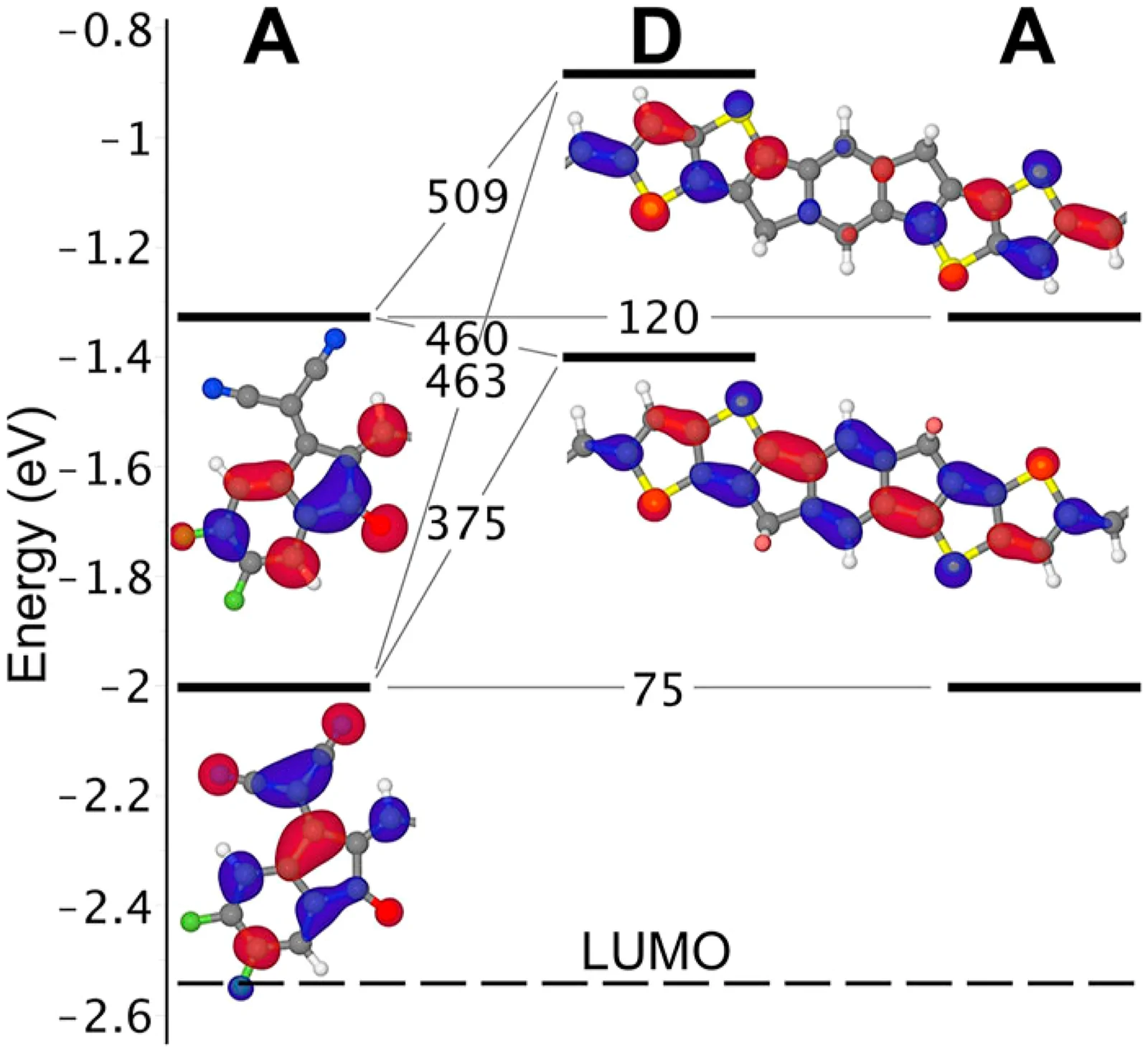
Coarse-grained model of the ITIC-4F molecule with two localized molecular orbitals (LMOs) per block. The LMO on the right side is the mirror image of that on the left-side acceptor. The coupling between the blocks is given as the electronic coupling in meV. The lowest LMO is more localized on the acceptor as compared to that of the 2-LMO model. This figure has been adapted/reproduced from ref. 112 with permission from AIP Publishing, copyright 2023.

## 5. Excited state of NFAs using DFT

The excited-state properties of NFAs are typically studied with TD-DFT, which gives detailed information about charge-transfer behavior, absorption spectra and electronic excited states.^114^ The electronic and optical properties of dithieno[3,2-*b*:2′,3′-*d*]silole-2,6-diyl as a donor molecule have been investigated by TD-DFT as well as by DFT calculations at the CAM-B3LYP/6-31G(d) level. The energies of these excitations, the reorganization energies, and the frontier molecular orbitals (FMOs) of the system were correctly calculated by these methods.^115^ Likewise, donor–acceptor (D–A) systems have also been studied at the B3LYP/6-31G level and shown potential for use in organic solar cells.^116^ In one such study, α-sexithiophene and dipyridophenazine were used as donor and acceptor materials, respectively, and the role of Ca, Mg and Be atoms in the photovoltaic performance of these materials was systematically investigated.^117^ TD-DFT has also been extensively used to study charge-transfer processes, and the larger the charge transfer, the more efficient the donor–acceptor interactions. In addition, by means of transition-density analysis, the excitation wavelengths as well as the pathways of excited-state transitions can be predicted.^118,119^ Ground-state and excited-state calculations using B3LYP/6-31G(d, p) have also been shown to be useful for computational studies of A–π–D–π–A molecular architectures such as those shown in Fig. 13, to predict band gaps, absorption characteristics, and photovoltaic efficiencies. TD-DFT has now become a key computational method to predict the absorption spectra, excited-state transitions, charge-transfer mechanisms and to aid in the molecular design of high-performance NFAs.^119^ It has been widely used to investigate electronic transitions and optical absorption of A–DA′D–A molecular structures, showing a wide absorption in the solar spectrum.^130^

**Fig. 13:**
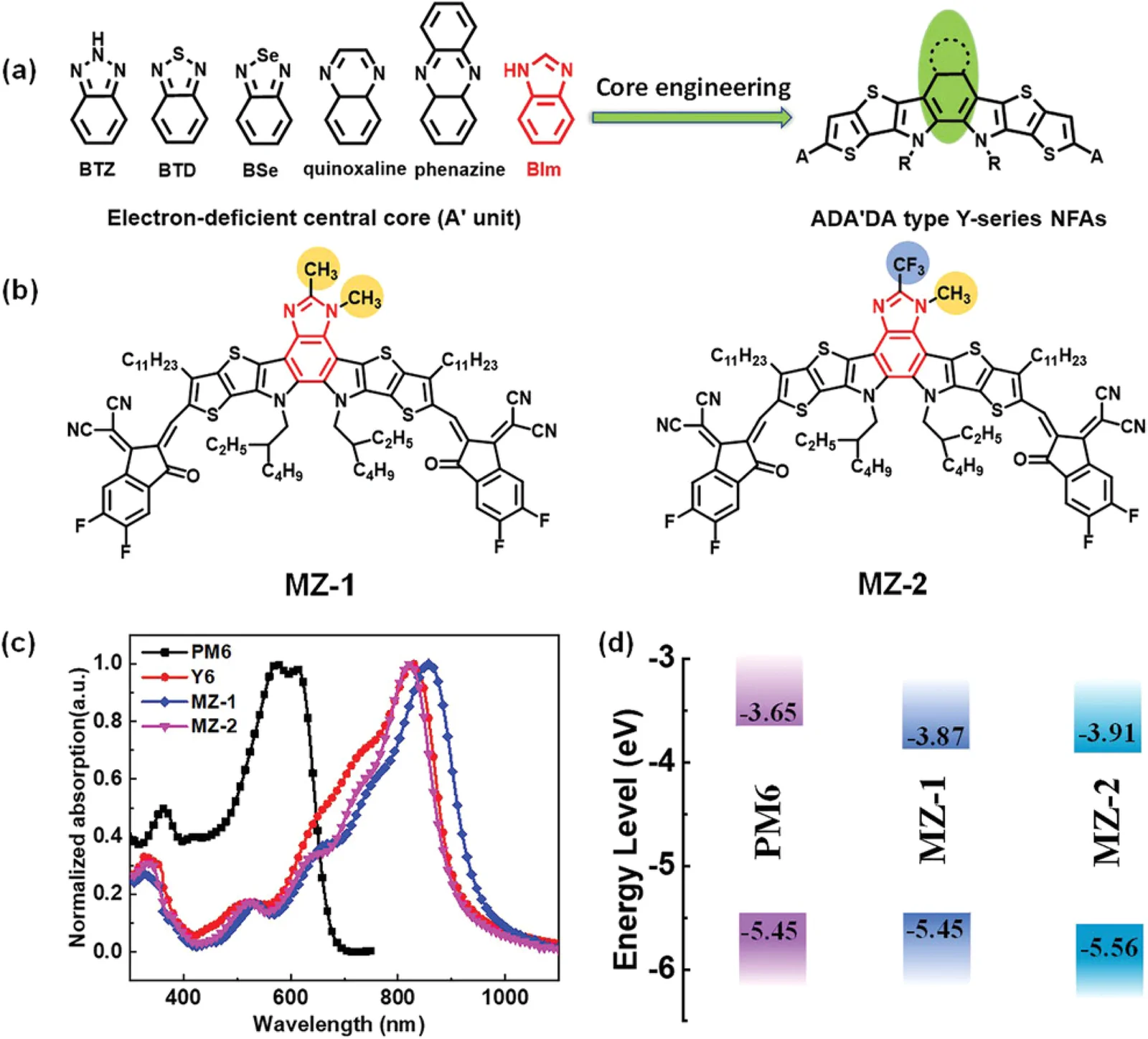
(a) The design concept of NFAs with an ADA'DA architecture; (b) the molecular structure of MZ-1 and MZ-2; (c) normalized UV-vis absorption spectra of PM6 and three NFAs in a thin film; (d) energy levels of PM6 and two NFAs. This figure has been adapted/reproduced from ref. 122 with permission from John Wiley and Sons, copyright 2023.

Hole–electron distribution analysis based on TD-DFT has also been employed to analyze the charge density in the excited states of Y6-type NFAs, in order to gain information about the charge-transfer mechanism for achieving efficient photovoltaic performance.^120^ The studies presented here show that the design of the molecule is very important for efficient charge separation and with respect to the reduction of charge recombination losses. The main strength of TD-DFT is that it provides static information about the excited-state electronic structure, whereas its combination with MD and other simulation methods allows the excited-state dynamics and charge-transfer processes to be studied in more detail.^59^ Therefore, excited-state calculations are useful to gain insight into both charge-transfer efficiency and charge-recombination behavior, which can be used to rationally design high-performance NFA materials.

## 6. Molecular dynamics simulations of NFAs

MD simulations connect the properties of individual NFA molecules with the morphology of the active layer, and they are therefore essential to understand molecular packing, charge transport and morphological stability in OSCs. In MD, the motion of atoms is followed by integrating Newton's equations of motion, and the interactions between atoms are described by a force field. The accuracy of MD simulations therefore depends critically on the force field. Several general force fields, such as GROMOS, OPLS-AA, CHARMM and GAFF, have been used for NFA systems.^131^ All-atom MD (AA-MD) simulations provide detailed information on local packing, whereas coarse-grained MD (CG-MD) simulations bridge the length and time scales to mesoscopic dimensions, in order to study domain formation, aggregation and phase separation in bulk heterojunction blends. The combination of AA-MD for local structure and CG-MD for global morphology has proved to be a successful approach to understand the structure–property relationships of NFA-based OSCs.^131^ More recently, machine-learning force fields have been developed, which approach first-principles accuracy at a much lower computational cost and are expected to improve the prediction of bulk and thin-film morphologies.^132^

### 6.1 Force-field refinement and generation of morphologies

General force fields are not parametrized for the extended conjugated backbones of NFAs. In particular, the torsional potentials between the fused core, the π-bridges and the end groups, which control the planarity of the molecule and hence its packing, are often poorly described. Therefore, these torsional potentials are usually re-parametrized against DFT scans of the dihedral angles, and the atomic partial charges are fitted to the DFT electrostatic potential. The morphology of the blend is then generated either by annealing a randomly packed system (heating above the glass transition and slow cooling) or by explicit simulation of solvent evaporation, which mimics film formation during spin-coating. From the equilibrated trajectories, the morphology is analysed quantitatively in terms of (i) radial distribution functions and π–π stacking distances, (ii) orientational order parameters describing face-on or edge-on packing, and (iii) the size and connectivity of donor and acceptor domains and the donor/acceptor interfacial area. Following these quantities as a function of time and temperature gives the morphological evolution of the blend, while the mean square displacement of the molecules gives their diffusion coefficients. These parameters are directly related to the thermal stability of the devices discussed in Section 9.

### 6.2 From MD trajectories to charge-transport parameters

The electronic properties of the simulated morphology are obtained by combining MD with quantum-chemical calculations.^133,134^ First, pairs of neighbouring molecules are identified in the MD snapshots, *e.g.* molecules whose conjugated segments lie within a cutoff distance. For each pair, the transfer integral (electronic coupling) *J*_*ij*_, which measures the overlap of the frontier orbitals of molecules *i* and *j*, is calculated. In the dimer-projection approach, the frontier orbitals of the isolated monomers are projected onto the orbitals of the dimer calculated by DFT, which gives *J*_*ij*_ without the need for symmetric dimer configurations.^133^ Since thousands of pairs are required, semi-empirical methods such as ZINDO are also used for screening. Second, the site energy *E*_*i*_ of each molecule is evaluated, including the electrostatic and polarization contributions of the surrounding molecules. The distribution of site energies gives the energetic disorder (*σ*) of the material. Third, the internal reorganization energy (*λ*) is calculated by DFT using the four-point scheme from the energies of the neutral and charged states at their optimized geometries. These parameters are combined in the Marcus expression, eqn (3), for the rate of charge transfer from molecule *i* to molecule *j*:^135,136^

3 *k*_*ij*_ = (2π/*ħ*) |*J*_*ij*_|^2^ (4π*λk*_B_*T*)^−1/2^ exp[−(Δ*E*_*ij*_ + *λ*)^2^/(4*λk*_B_*T*)]

where Δ*E*_*ij*_ = *E*_*j*_ − *E*_*i*_ is the difference in site energies, *k*_B_ is the Boltzmann constant and *T* is the temperature. Finally, the rates obtained from eqn (3) for all pairs are used in kinetic Monte Carlo simulations of charge hopping through the morphology, from which the charge-carrier mobility and its dependence on the electric field and charge density are obtained.^134^ Because the couplings fluctuate with the thermal motion of the molecules, they are averaged over several MD snapshots, and the distribution of *J*_*ij*_ gives the dynamic disorder.

For NFAs, this workflow has shown that the three-dimensional packing of Y-series acceptors through their end groups creates multiple coupling pathways, which delocalize the exciton and electron wavefunctions.^137^ Similarly, DFT-based estimation of transfer integrals and reorganization energies has predicted up to a four-fold increase in electron mobility for modular non-fused derivatives of BTP-4Cl.^138^

### 6.3 Case studies

An interesting case study of the power of MD simulations is the blend system used for PM6:Y6. Based on the *ab initio* molecular dynamics (AIMD) simulations, it has been found that the donor and acceptor molecules in PM6:Y6 films show π–π stacking with ordered arrangement of Y6 molecules. The simulations found the exciton binding energy (*E*_b_) of the PM6:Y6 system to be less than 0.1 eV and the singlet to charge transfer energy gap (Δ*E*_S1–CT_) to be less than 0.1 eV, which is very favourable for an efficient charge separation. Moreover, the Marcus theory calculations, using configurations deduced from the MD simulations, yielded high electron and hole transfer rates, up to 10^12^ s^−1^ for charge transfer at the donor–acceptor interface in the case of exciton dissociation. These computational results are consistent with the experimental ones, thus confirming the ability to predict charge generation/recombination processes in high-efficiency OSCs. According to MD simulation, π–π interactions between the donor and acceptor molecules can enhance the number of heterojunction interfaces which can promote charge transport. Moreover, the interfacial area can be increased and charge transfer can be facilitated by hydrogen bonding between certain functional groups.^139^ The morphology and charge-transport properties of polymer NFA blends have also been extensively studied in these simulations, where increased intermolecular interaction leads to better heterojunction formation and segregation and thus better performance for polymer:NFA blends with different NFAs.^130^ The interfacial processes which can be described by combining MD simulations with DFT calculations are illustrated schematically in Fig. 14. The packing of NFA molecules can be greatly influenced by slight differences in structure, *e.g.* side chains and degree of planarity, and the more planar molecules are more likely to form ordered π–π stacking arrangements, which lead to good charge transport.^140,141^

**Fig. 14:**
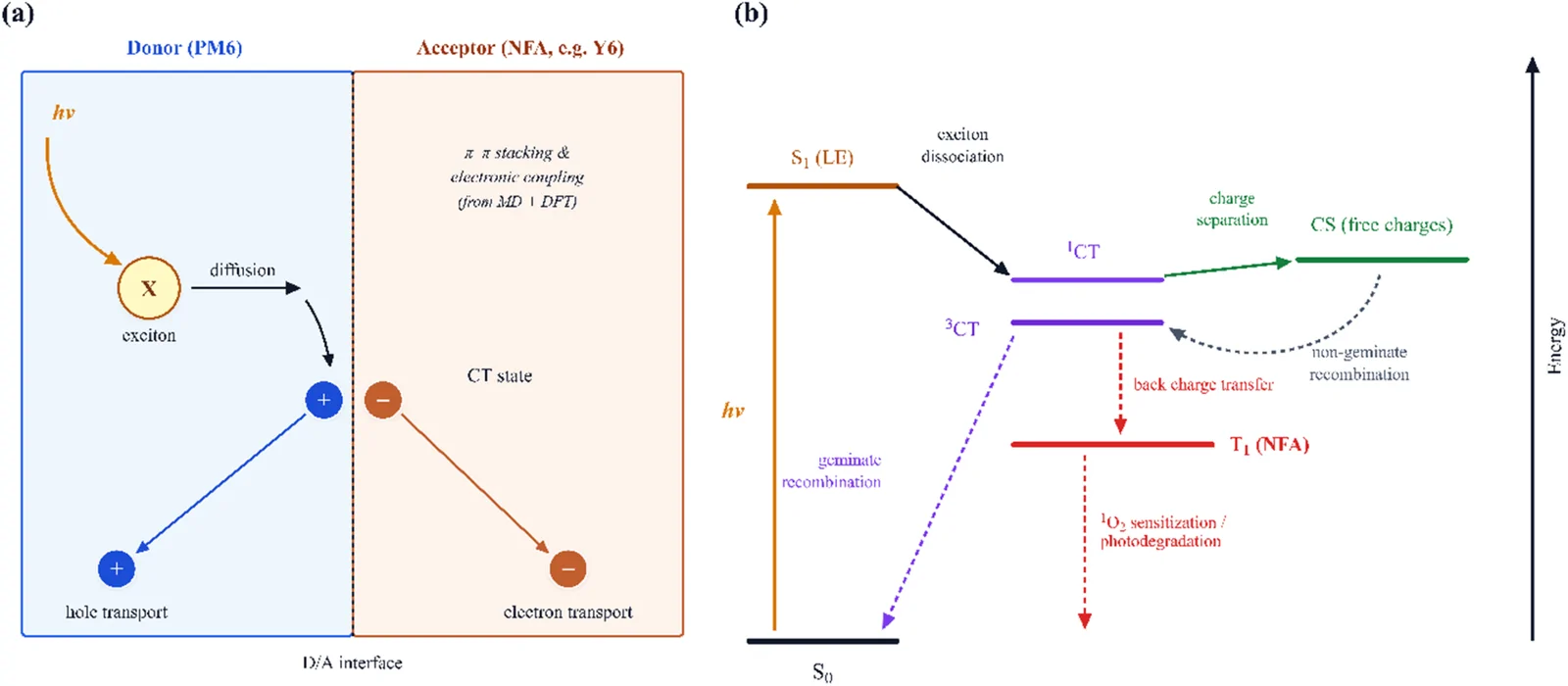
Schematic illustration of (a) the processes occurring at the donor/acceptor interface, *i.e.* exciton diffusion, formation of the charge-transfer (CT) state and charge separation, where π–π stacking and electronic coupling can be obtained from MD simulations combined with DFT, and (b) the corresponding state-energy diagram showing charge separation and the main loss pathways, including geminate and non-geminate recombination, back charge transfer to the NFA triplet state (T_1_) and triplet-sensitized singlet-oxygen formation that leads to photodegradation.

MD simulations have proved to be quite useful in understanding NFA morphology control by solvent additives. In the PM6:Y6 system, simulations have indicated that the additive 1-chloronaphthalene (1-CN) has a dual effect: it reduces over-aggregation of Y6 and at the same time it enhances the crystallinity inside the domains. This double action increases the charge transfer between neighbouring Y6 molecules and PM6 chains.^142^ Notably, the PM6:Y6 blends prepared in a variety of solvents assemble into different structures and morphologies, which is a key factor in device performance since the relative orientation of the Y6 sidechains and end groups to the fused-ring cores plays a crucial role in dictating device performance. All-atom MD simulations have also revealed that the short-range aggregation of Y6 molecules is a natural morphology which facilitates charge extraction and reduces charge recombination.

## 7. Machine learning in designing NFAs for OSCs

ML is a branch of artificial intelligence (AI) that uses statistical and mathematical algorithms to find useful information in large sets of data.^143^ It can help identify correlations and complex relationships that may not be apparent from traditional analysis. Due to its predictive potential, ML has found widespread use to study current and novel materials that exhibit desired functions. As a result, numerous research papers, reviews, and perspective papers have emerged on the applications of ML in materials science. It has recently been shown that the design of NFAs using ML is much more efficient than the conventional trial-and-error synthesis. Therefore, ML has emerged as an effective computational tool for the discovery and optimization of NFAs for OSCs. The molecular structures and their related properties, such as energy levels, light-harvesting ability, and charge-carrier mobility, can be predicted using ML algorithms.^144^ This predictive modelling can greatly decrease the cost and time needed to identify potentially successful NFA families. Furthermore, ML enables the understanding of complex structural relations that cannot be easily conveyed by manual analysis. After the molecular properties are predicted, NFAs exhibiting optimized optoelectronic properties can be rationally designed by optimizing the energy-level alignment and the absorption spectrum. The approach is contributing to the creation of very efficient and stable PV materials. ML includes a wide variety of algorithms which learn from the given data and make predictions without explicit programming. The ML strategies which have been used for the design and optimization of NFAs are discussed below.

### 7.1 Supervised learning

A machine-learning method that requires the input variables (*e.g.*, molecular and electronic descriptors) and the output values (*e.g.* PCE) to be available during model training to reliably predict material properties is called supervised learning (SL).^145^ SL has been extensively used in NFA research to estimate charge-transport parameters, optical parameters, frontier energy levels, band gaps, and photovoltaic parameters. Most often, the design of new NFAs based on supervised learning is carried out in four major steps. First, data sets are compiled that include the experimentally determined NFAs along with the molecular descriptors such as energy level, dipole moments, functional groups, and the conjugation length for each NFA, as shown in Fig. 15. Then, ML algorithms, including support vector machines (SVMs), neural networks and random forest models, are trained to set up the relationship between the molecular descriptors and the properties of the target material. At this point, the model learns from the given set of input–output pairs and adapts its internal parameters to reduce the prediction error. Then, to assess the reliability and generalization of the trained model, validation techniques such as *k*-fold cross-validation are used. Lastly, the optimized model can be used to predict the properties of new and untested NFAs and help design new materials with desirable properties for OPV applications.^146^ More data in supervised learning tends to improve the accuracy of predictions, whereas smaller data sets result in less accurate predictions.^147^

**Fig. 15:**
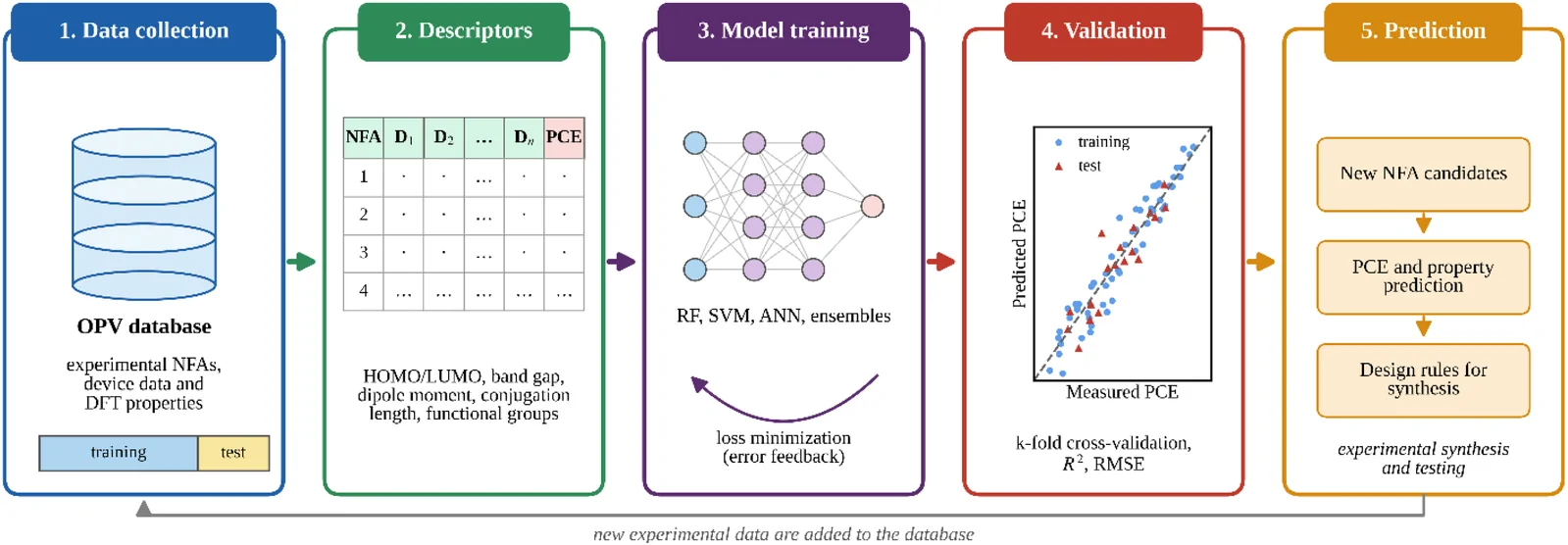
Workflow for the prediction of molecular descriptors and material properties from a large NFA dataset with a supervised ML model.

A random forest algorithm was trained with the molecular descriptors (HOMO/LUMO energy levels and absorption spectra) as input variables to predict the PV performance of about 200 NFAs.^148^ The trained model was able to predict the structural motifs that have high PCE values and predict promising NFAs for OSC applications. Likewise, supervised learning has been used to estimate charge mobility, using SVM algorithms that correlate molecular descriptors with charge-transport behaviour. The molecular size, π-electron donating and electron withdrawing substituents, and the length of π-conjugation are important parameters that determine charge mobility in the NFA molecules.^149^ The trained SVM model showed good performance in predicting the mobility of previously unexplored NFAs, which helped to optimize charge collection in solar cells. In addition, artificial neural networks have been used to predict the band gaps of NFAs, which gives information about charge separation and efficiency of light absorption in photovoltaic devices.^150^

Supervised learning has also been used to design NFAs with both low and high band gaps along with wide absorption spectra. Although SL models make very good predictions, they depend strongly on high-quality experimental data. Furthermore, some ML algorithms can be complicated, compromising model interpretability, which can make it difficult to identify the key molecular properties contributing to the predicted photovoltaic properties.

### 7.2 Unsupervised learning (UL)

Unsupervised learning (UL) is an ML method to discover hidden patterns in datasets without any labelled output patterns.^151^ In contrast to supervised learning where input and output information are known during the learning process, UL learns relationships among input data based on the data's intrinsic similarities. This is very helpful when clustering together materials that have similar structural and electronic properties and is useful for understanding the factors that limit the performance of photovoltaics. In NFA research, UL has been used for molecular clustering, screening, and optimization of the materials based on their physicochemical properties. First, molecular descriptors like electron affinity, conjugation length, and dipole moment are collected. Dimensionality-reduction techniques, such as principal component analysis (PCA), are then used to reduce the number of variables in the dataset while keeping the important information. The materials with similar characteristics are then clustered by various methods, such as K-means clustering, such that families of NFAs with similar charge-transport behaviour, optical properties, and molecular structures can be identified. These types of analyses can aid in the identification of new NFAs with desired absorption and charge-transport properties.^152^ UL has enabled researchers to gain a much deeper understanding of the complicated molecular interactions that are hard to uncover with traditional methods. For instance, materials have been successfully classified by using their molecular descriptors by applying the K-means clustering method, and thereby the effect of similarity on the photovoltaic properties of materials has been studied. Similarly, hierarchical clustering of NFAs according to molecular properties showed that the A–D–A molecules tend to have higher PCE values.^153^

### 7.3 Reinforcement learning (RL)

Reinforcement learning (RL) is a powerful ML technique which enables an intelligent agent to acquire the best decision-making policy by continual interaction with the surrounding environment. In contrast to supervised learning, which relies on labeled data sets, RL learns by experimenting with actions and gradually fine-tunes future actions based on the feedback it receives. This learning strategy is especially useful in the case of no labelled output data: the model is created by repeatedly exploring and exploiting the search space and provides optimal solutions. Recently, RL has also been used for the design of NFAs, showing great potential in optimizing molecular structures and enhancing the performance of photovoltaics. The main goal of RL is to discover materials with design features that enable the maximum efficiency of OSC by systematically optimizing molecular parameters like energy level and molecular size. This is a process that is repeated, and feedback on the molecular morphology and electronic structure, typically acquired from DFT calculations, is utilized for further modification of the structure. The molecular characteristics, such as light absorption, electron mobility, donor–acceptor interaction, and π-conjugation, are optimized continuously to search for molecules that have potential as NFAs.^30^ One such case that has been mentioned in the literature used a deep Q-network to design NFAs with better optical properties and better charge transfer characteristics.^154^ This framework led to the creation of novel molecular architectures, as well as optimization of donor–acceptor interactions and π-conjugation, leading to materials with enhanced predicted photovoltaic properties. The results in this work show the ability of RL to explore previously under-explored areas of chemical space and to find potential molecular designs. Despite these benefits, RL is computationally intensive, especially for large datasets, and can only be applied with significant feedback and high-quality data, which are hard to obtain in the case of limited experimental data.^155^

In order to enhance performance and optimize the design of NFAs for solar cells, the combination of SL for the prediction of properties and RL for describing molecular design has proved to be a more comprehensive method. This hybrid technique can enhance the optimization of NFAs, explaining their optical properties and PCE.^156^ Moreover, RL provides feedback from the predictions, which helps to enhance the properties of the materials. In such a hybrid method, the optical properties have been predicted by SL, whereas RL has been used to explore unseen molecular structures with high PCE. Resultantly, the hybrid approach not only provides new NFA molecules but also predicts the light absorption and charge transfer efficiently. ML is a powerful tool to design NFAs for solar cells, as it helps to predict the properties and optimize the structure.^37^ The key applications of ML in designing NFAs are optimization and prediction of properties, screening of molecules and structure–property relationships. These approaches help to discover and tune the properties of NFAs, and to enhance the performance of OSCs.

The most effective application of ML is its ability to predict properties like charge transport, PCE and optical absorption, key features for high-performance solar cells. By using ML models, researchers can easily predict properties of new materials which reduces the time needed for experimental work.^157,158^ By using descriptors like bandgap, molecular weights and electron density, ML can predict the PCE of different types of NFAs. The random forest model predicts the PCE of NFAs like Y6 and ITIC with higher accuracy, which is further used for testing and designing materials for more efficient OSCs. The charge transport properties of NFAs like IDIC and IT-4F can be predicted by neural networks, which consider properties like molecular planarity and alkyl substitutions.^151^ The structural properties of larger datasets can also be predicted by using ML models.^159^ The relationship between PCE and molecular structures can be predicted by using SVM models and decision trees. The features like charge transfer and morphology of materials like PM6:Y6 and PBDB-T:ITIC can be determined, highlighting the significance of conjugation and side chain properties of OSCs.^160^

### 7.4 Generative models for NFA design

Recent advances in deep learning have enabled the design of new molecules with desired structural and electronic properties through generative models such as variational autoencoders (VAEs), GANs and convolutional neural network (CNN) based frameworks. These models reduce the reliance on repeated synthetic trials.^161^ In contrast to the conventional route of molecular development, which involves synthesis followed by experimental testing and evaluation, generative models learn the relationship between structure and property from existing data. They then create new molecules which meet certain design criteria, *e.g.* suitable energy-level alignment and the desired optoelectronic properties. In this way, the development cycle is shortened, and molecular architectures which are not easily accessible by traditional methods can also be explored.

One representative example is a GAN-based framework in which molecular structures and their corresponding physicochemical properties were utilized to train the model.^162^ It has been reported that such a framework generates new A–D–A type structures with improved charge transport and light absorption, and a modified NFA (F1) with improved efficiency was obtained in this way. Similarly, a variational autoencoder has been used to explore the chemical space of NFAs, with the main focus on optimizing the electron-deficient sites and the planarity of molecules, which are crucial for charge transport and energy-level adjustment. By using this approach, new NFAs like IT-M and PCPDTBT-IC with promising efficiency could be predicted. In addition, a CNN-based generative design framework has been used to create NFAs having desirable energy-level alignment and strong absorption in the visible region.^151^ With this strategy, the molecular structure F16BT-C6 was designed, and it showed good photovoltaic properties along with better light-harvesting capability.

### 7.5 DFT-ML integrated screening and prediction of absorption

DFT coupled with ML has emerged as an effective method to screen potentially good NFAs on the basis of their electronic properties. Researchers can use the computational methods coupled with predictive ML algorithms to assess electronic properties, energy levels, and PV functionality prior to experimental synthesis. The integrated workflow can drastically speed up the discovery of high-performance NFAs as shown in Fig. 15. In a computational screening study, over 3000 molecular candidates have been screened as potential NFAs.^37^ The band gaps and electronic and optical properties of the screened molecules were predicted using a random forest regression model and DFT calculations. On the basis of these predictions, two promising NFAs, namely IQIDIW02 and PHTLBC02, were selected for synthesis and then tested in OSCs.^55^ The combination of deep learning and DFT calculations was also used in another important investigation to develop molecular descriptors for new NFA molecules that predict their structure and PV performance. This computational scheme was able to identify the compounds C6TTF and TT-TPE, which were both confirmed and tested, with improved device performance and PCE. These studies show how much ML combined with DFT can help accelerate material discovery and aid in the identification of high performing families of NFAs.^163^

Two main types of data are used to construct machine learning models in materials science. The first type includes datasets generated by DFT, including high throughput computational data like optimized molecular geometries, band structures and electronic properties derived from first-principles calculations. The advantage of these datasets is that they can be used to investigate large chemical spaces in which there is little experimental data. The second class consists of experimentally measured data, such as device efficiencies, degradation behavior and other PV properties representative of the actual device performance behavior of material. These computational and experimental data can be used together by ML models to build reliable structure–property relationships.

It is important to measure a material's light-harvesting ability, which directly affects the PCE of organic solar cells. Traditionally, the absorption characteristics were obtained through experimental measurements or simulation methods involving a high level of computing. Recently, ML has also offered an efficient solution, quickly predicting absorption based on large set of molecules. The structural descriptors that can be used for designing ML models comprise molecular symmetry, conjugation length, electronic charge distribution, *etc.* which can distinguish the structures with high absorption ability over the entire solar spectrum from others with low absorption to possess high photovoltaic performance. Using 500 conjugated molecules, ML has been used to predict the strength of absorption of NFAs. To determine the extinction coefficient of those molecules indicating the strength of their absorption, the TD-DFT calculations have been initially carried out.^164,165^ These computed descriptors were then used to train a random forest regression model, which was able to correctly predict the extinction coefficient and structure of molecules not included in the original training set. This method discovered NFA-BT-1 that had an excellent absorption power at 800 nm in the near-infrared region, and thus improved the solar cell efficiency. In another study, the authors investigated prediction of the absorption spectra of the NFAs using SVM models on large molecular datasets.^166^ The developed model was able to identify the promising materials, including PCPDTBT and DTPD which exhibited good absorption properties in OSC. The excellent correlation between the computed and experimental results, further, confirmed the reliability of ML as a tool to assess the optical properties of emerging NFA materials.

### 7.6 Extrapolative ML for efficiency prediction

Through extrapolative ML models, a large number of experimental absorption spectra, charge-carrier mobilities and frontier energy levels are used to predict the PCE of OSCs. The models are especially useful when they can be used to estimate performance of materials that have not yet been tested in experiments, thus reducing the need for experimental screening and providing an efficient way to identify potential materials for experimental study.

Extrapolative ML has been shown to predict the efficiency of ternary OSC systems based on a compilation of nearly 300 OSCs tested in the lab with experimentally known HOMO/LUMO energy levels and device efficiencies. The performance of the BTR-Q_*x*_ system was predicted with good accuracy using a random forest model. The predicted PCE of about 13% was then verified with experimental measurements, illustrating the accuracy of the efficiency prediction using ML. Similarly, another study used a gradient boosting model to predict the PCE of ternary systems. In this way, a good photovoltaic material, the NFA M-CN, with a predicted PCE of 14%, was chosen and its viability was verified by experimental measurements, which showed a PCE of 14.5%.^146,167^

ML is a powerful method to effectively accelerate the discovery of materials in polymer-based organic solar cells, including the discovery of new high-performance NFAs. The process of discovering a new material is conventionally very tedious and resource intensive, involving the repeated synthesis, characterization and device fabrication.^168^ By contrast, ML facilitates the quick prediction of crucial molecular traits such as optical, electronic and charge-transport before experimental synthesis, as shown in Fig. 16. Additionally, ML can help to identify molecules in large molecular libraries with attributes desirable for high efficiency polymer solar cells. Another successful application of ML has been in the identification of promising donor materials to incorporate in fullerene- and polymer-based solar cells.^169^ Using the existing molecular data and photovoltaic performance data, a predictive model was trained and the donor material IDT-BT was found to have a PCE of nearly 13%. The use of ML has now been extended to tandem solar cells, in which predictive models aid in identifying materials that can offer better device performance.

**Fig. 16:**
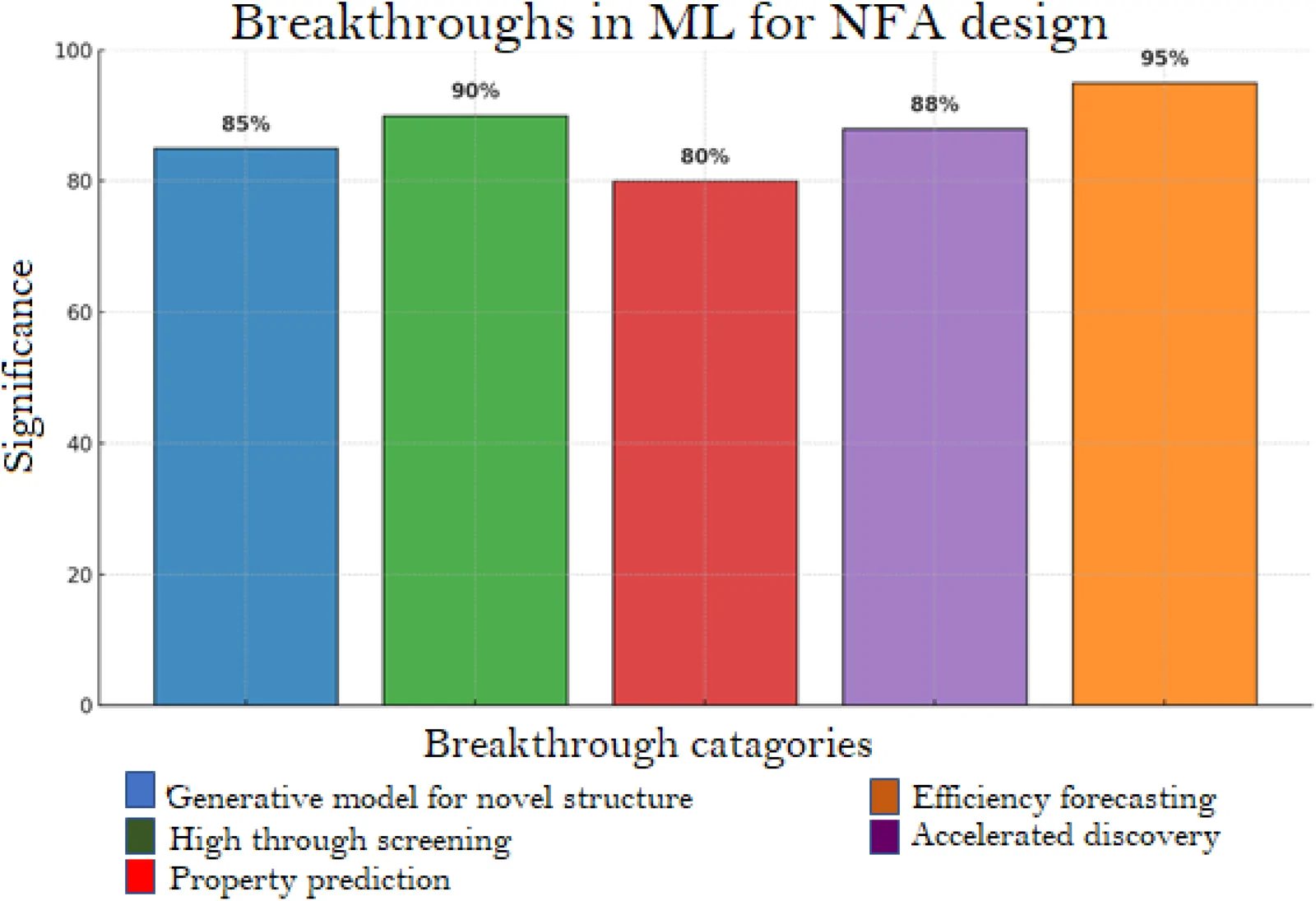
Breakthroughs of ML in designing NFAs on the basis of different properties and their significance.

Targeted molecular descriptors were, for instance, used in the training of the ML models for the identification of materials with high photovoltaic performance, as in the identification of the material TTBT-2.^170^ The results show that the use of ML is a promising way to search for novel NFAs that possess desirable properties. Furthermore, its applications are not limited to single-junction solar cells but also extend to ternary and tandem OSCs, which has significantly shortened the material development time compared to the typical experimental procedure.^171^

### 7.7 Challenges and limitations of ML

Despite the success of ML in the design of NFAs, several challenges limit the reliability of the reported predictions, and these should be considered critically.

The first challenge is the scarcity and heterogeneity of experimental data. Compared with other fields of materials science, the number of experimentally tested OSC materials is small. For instance, the Harvard organic photovoltaic dataset contains 350 molecules with experimental device data, along with the corresponding DFT-calculated properties.^159^ Moreover, the reported PCE of the same donor:acceptor pair varies with device architecture, processing and morphology, which introduces noise that is not related to the molecular structure. Large computed datasets are available, but the properties calculated for isolated molecules do not capture the morphology-dependent device performance.

The second challenge is the representation of molecules. SMILES strings and 2D fingerprints are easy to generate, but they do not contain information on molecular conformation, planarity or packing, which are crucial for Y-series acceptors. Moreover, different SMILES strings can describe the same molecule, and similar fingerprints can belong to molecules with different backbone conformations. 3D conformer-based descriptors and physically meaningful descriptors derived from DFT, *e.g.* frontier orbital energies, reorganization energies and charge-transfer character, provide better performance for small datasets,^157,158^ but they require additional computational cost and depend on the chosen conformer.

The third challenge is data leakage and inflated accuracy. When molecules sharing the same core or end groups are distributed randomly between the training and test sets, the model is effectively tested on molecules it has already seen, and the reported accuracy is overestimated. Therefore, scaffold-based or time-based splitting of the data should be used, and the ML model should be compared with simple physical baselines, *e.g.* the Scharber model based on frontier orbital energies, to demonstrate a real improvement.

The fourth challenge is out-of-distribution generalization. The most interesting candidates for discovery are, by definition, different from the known materials, whereas ML models interpolate most reliably within the chemical space of their training data. Models trained mainly on fullerene-based or early NFA systems are thus not expected to predict the performance of new acceptor families accurately. Extrapolative models should therefore report their domain of applicability and the uncertainty of their predictions, *e.g.* by using Gaussian-process regression or ensembles of models.^147,170^

Several strategies are emerging to address these challenges: active learning, which selects the most informative molecules for synthesis or calculation; transfer learning from large computed datasets to small experimental ones; physics-informed descriptors; and explainable ML methods which identify the structural features behind the predictions.^145^ Moreover, the inclusion of stability data, which is still very scarce, is essential if ML is to be used for designing NFAs with long operational lifetimes.

## 8. Breakthroughs in designing the NFAs

The computational methods are essential to accurately predict the morphology, electronic structure and photovoltaic properties of NFAs with high reliability.^172^ These methods can be used to screen a range of materials without relying solely on experimental synthesis by examining their absorption properties, the behaviour of charge transfer and their electronic properties using DFT based methods. DFT calculations can also be used to give detailed information on the electronic structure, HOMO/LUMO energy levels, molecular stability and charge-transport properties of candidate materials.^173^ As a result, computational design has contributed greatly to the optimization of molecular structures and engineering of morphologies for improving the efficiency of organic photovoltaic devices. It is shown in Fig. 17 that the computational screening strategy of A–D–A-based NFAs involves a series of iterations.

**Fig. 17:**
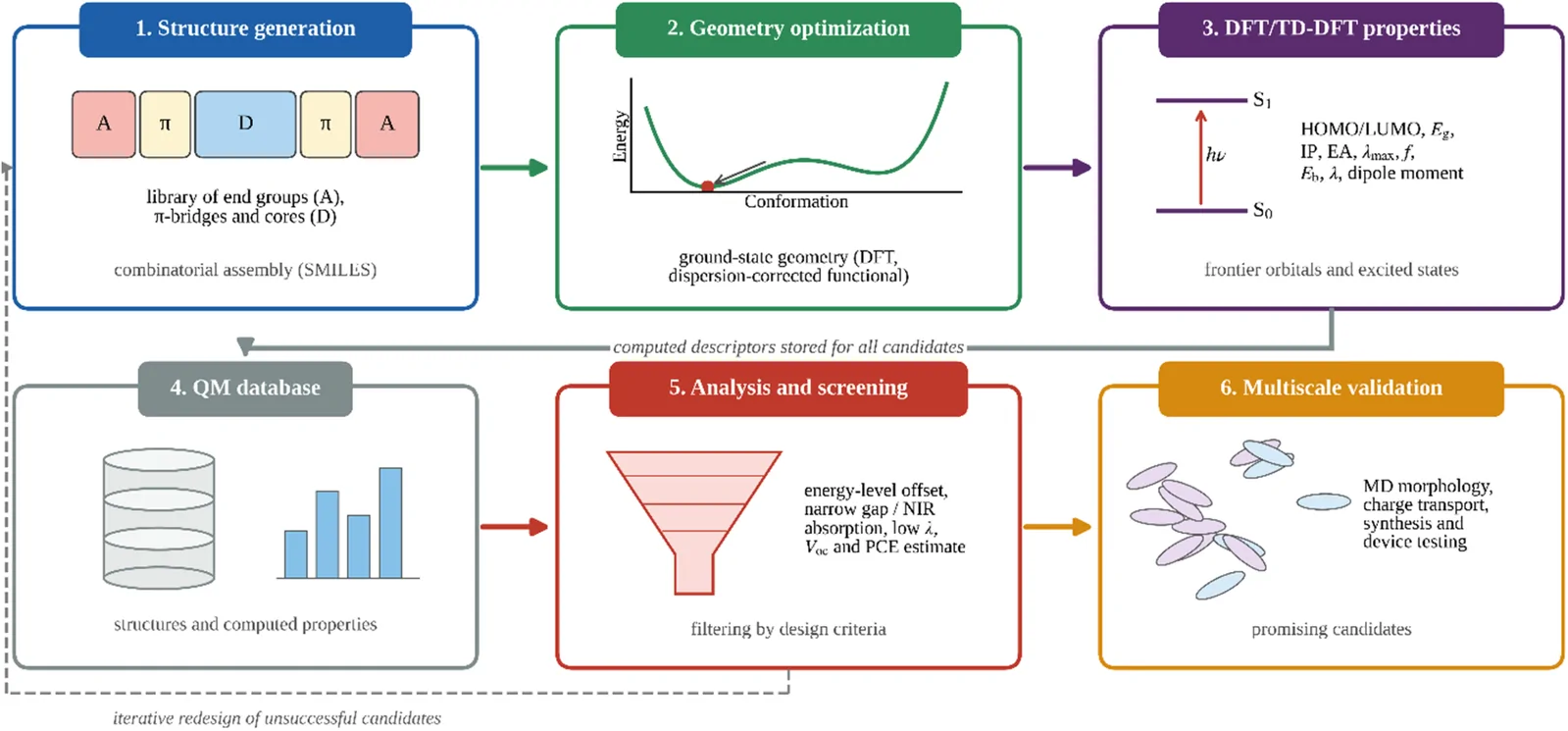
Computational screening strategy for designing A–D–A-based NFAs using DFT.

### 8.1 From ITIC to Y-series acceptors

One of the major breakthroughs in the area of computer-assisted NFA design is the creation of Y6, a high performance non-fullerene acceptor that has greatly enhanced the efficiency of OPVs.^174^ Y6 was developed on the basis of the design principles of A–D–A acceptors such as IT-4F, and computational studies have been used extensively to understand and improve its efficiency. The DFT calculations have been useful to understand its optical properties, molecular geometry, and electronic structure. The optimized energy levels of Y6 match well with those of the donor polymer PM6, which makes the charge transfer between the donor and acceptor components easy. Furthermore, fluorination and further π-conjugation shift the absorption spectrum to the near IR region. Consequently, the PM6:Y6 blend has received much interest and has a PCE of over 16% which is higher than several fullerene-based blends.^175^ Computational studies are largely responsible for these improvements, as they helped to understand the interplay between molecular structure/geometry, morphology and the photovoltaic performance.

The combination of ITIC with the donor polymer PBDB-T provided a PCE of over 11% along with an excellent thermal stability.^176^ ITIC has been extensively used in DFT studies to see how molecular modification affects its electronic behavior. The introduction of EWGs in the ITIC structure, in particular, decreases the LUMO energy level and increases the EA and planarity of the molecule.^175^ One of the other significant improvements is the computational design of BTP-4Cl and BTP-4F, where DFT has been employed to optimize the molecular structures.^142^ More recently, DFT has also been used to deconstruct the fused backbone of BTP-4Cl into modular non-fused A–π–A′–π–A acceptors, in order to reduce the synthetic complexity of Y-series acceptors. It has been observed that the designed acceptors maintain the narrow energy gaps and near-infrared absorption of the Y-series, while showing lower reorganization energies, up to a four-fold higher electron mobility and stronger electronic coupling with the PM6 donor when compared to BTP-4Cl.^138^ These acceptors are based on the fused benzothiadiazole–thiophene–pyrrole (dithienothiophen[3,2-*b*]pyrrolobenzothiadiazole) core, and exhibit a broad visible and near-infrared absorption and enhanced charge transport properties. Using DFT studies, it was also shown that the EA of BTP-4F increases with fluorination while the band gap decreases, which favors charge transfer and light absorption, respectively.^177^ Similarly, the frontier energy levels can be adjusted by chlorination and donor–acceptor energy matching can also be enhanced. These acceptors, in combination with PM6, reached a PCE of almost 16%, highlighting the positive impact of their halogen substitution on PV performance.

### 8.2 DFT in simulating morphology and electronic properties

Besides predicting frontier energy levels, DFT is also important to interpret the morphology and charge transport behavior of molecules, which are crucial for the performance of NFA-based organic solar cells. In circular Y-series acceptor derivatives, favorable optoelectronic properties, such as relatively high-lying LUMO energy levels, reduced band gaps and excellent electronic coherence between the central core and terminal acceptor units, have been found in DFT investigations.^178^ These features make the materials more likely to have low reorganization energies and a more extensive absorption spectrum, offering novel opportunities for the development of high-performance NFAs.^179,180^

### 8.3 Recent systems beyond 20% efficiency

With the fast development of organic solar cells in recent years, the PCE has passed the 20% mark, and computational techniques are increasingly becoming a key element in the design and optimization of desired molecules. Several case studies are given below, highlighting the results and contributions of DFT, MD, and ML approaches in discovering state-of-the-art NFA materials with record efficiencies over 20%. Table 4 presents a summary of selected high-performance NFAs developed through computationally guided design and the specific computational strategies used, and their significant quantitative contributions to device performance.

**Table 4:** A summary of the computational methods employed for NFAs and their key contributions to device performance. ESP: electrostatic potential

NFA system	PCE (%)	Computational method	Key computational insight	Year	Ref.
Ternary PM6:PBTQ-Cl:BTP-eC9	20.3	DFT + ESP	Halogenation lowers HOMO; lowest dipole moment gives better miscibility and stability	2026	181
Ternary PM6:BTP-eC9:*o*-BTP-eC9	19.9	DFT	41 meV energy loss reduction	2024	92
Asymmetric side-chain acceptor	20.1	DFT + MD	Packing optimization; enhanced mobility	2025	93

More recently, a halogenation strategy has been applied to design scalable wide-bandgap polymer donors PBTQ-F, PBTQ-Cl and PBTQ-2F using theoretical and experimental methods.^181^ DFT calculations carried out at the B3LYP/6-31G(d,p) level of theory revealed highly planar polymer backbones with dihedral angles below 2°. Further, it has been observed that the halogen substitution systematically downshifts the HOMO/LUMO levels. The calculated values of dipole moments followed the order PBTQ-Cl (3.91 D) < PBTQ-F (4.84 D) < PBTQ-2F (5.02 D), and the lowest dipole moment of PBTQ-Cl appeared linked with its better miscibility with the BTP-eC9 acceptor and a more stable blend morphology. The binary device PBTQ-Cl:BTP-eC9 delivered a PCE of 18.55% with exceptional thermal stability. The use of PBTQ-Cl as the third component in PM6:BTP-eC9 appeared to reduce the π–π stacking distance from 3.758 to 3.737 Å and to increase the crystal coherence length from 14.69 to 15.11 Å. Resultantly, the PCE of the ternary device has been reported to increase from 18.87% to 20.30% (*V*_oc_ = 0.874 V, *J*_sc_ = 28.84 mA cm^−2^, FF = 80.52%), along with a lifetime of over 4000 h at 85 °C.^181^ This work predicts that the trends in energy levels and dipole moments can help in designing efficient and stable donor materials.

Another breakthrough is related to ternary system PM6:BTP-eC9:*o*-BTP-eC9, which shows the worth of DFT-based molecular design. Theoretical calculations allowed the rational optimization of the molecular structure, which predict an energetically favourable charge-transfer state and a very low energy loss of 41 meV in comparison to the parent BTP-eC9 molecule. The ternary system attained a PCE of 19.9% with better operational stability. This work sheds light on the power of DFT calculations to systematically guide molecular engineering for fulfilling multiple key requirements. The requirements include a weakly absorbing CT state, a high dielectric constant, a suitable surface energy and proper crystallinity.^92^ It should be noted that the molecular packing predicted by such calculations can be realized only when the film morphology is properly controlled during processing. For example, off-centre spin-coating has been used to apply a strong shear force during film formation, which induced a face-on orientation of the high-molecular-weight D18 donor, reduced the π–π stacking distance and increased the crystal coherence length. Consequently, the D18:L8-BO device appeared to provide a PCE of 20.1% with an FF of 81%, and the ternary D18:L8-BO:BTP-eC9-4Cl device achieved a PCE of 20.5%.^182^ These experimental results provide useful benchmarks for experimental validation of the predicted morphology.

A non-fullerene acceptor was molecularly designed and precisely engineered to achieve single-junction OSCs with 20.1% PCE. The alkyl side chains on the thiophene unit were optimized and made asymmetric with respect to the backbone to improve the BHJ morphology. As a result of the asymmetric design of the side chain, the π–π stacking distance was decreased from 3.58 Å to 3.52 Å, along with a switch in the electronic coupling from H-type to J-type, which was also supported by calculations. This change at the molecular level led to an improvement in the charge transport mobility (from 1.2 × 10^−4^ to 3.8 × 10^−4^ cm^2^ V^−1^ s^−1^) and to a decrease in non-radiative recombination losses.^93^

### 8.4 Polymerized acceptors and multicomponent active layers

Besides small-molecule acceptors, the polymerization of Y-series small-molecule acceptors has emerged as an effective route to all-polymer solar cells (all-PSCs), which combine the strong near-infrared absorption of Y-series acceptors with the mechanical robustness and morphological stability of polymers.^183^ Computational methods have played an important role in understanding these systems. For instance, DFT calculations at the B3LYP-D3 level showed that replacing the thiophene linker with bithiazole in the polymer acceptor PY-BTz reduces the backbone dihedral angle from more than 11° to about 1°, owing to S⋯O non-covalent conformational locking and reduced steric repulsion. The more coplanar and ordered backbone delivered PCEs of 16.4% in spin-coated and 14.7% in blade-coated devices.^184^ MD simulations further revealed that polymerized Y6-type acceptors are more miscible with the PM6 donor than their small-molecule counterparts, with about 22% more donor–acceptor contact pairs. This leads to donor–acceptor percolation at the interface, which prevents the formation of acceptor aggregates and suppresses the burn-in degradation under illumination.^185^ More recently, oligomeric acceptors have been designed to combine the advantages of both classes. A linear tetramer acceptor (4Y-BO) with the same skeleton as the polymer acceptor PY-BO achieved a certified PCE of 19.58% (champion 19.75%) with PM6, as compared to 15.66% for PM6:PY-BO, along with a T80 lifetime (the time taken to reach 80% of the initial PCE) of 2125 h at 80 °C. DFT calculations showed that the tetramer has a lower internal reorganization energy and stronger intermolecular interactions, with a complexation energy of −39.92 kcal mol^−1^ as compared to −35.50 kcal mol^−1^ for the polymer.^186^

Multicomponent (ternary and quaternary) active layers are now the main route to efficiencies above 20%. In these systems, a guest acceptor which is structurally similar to the host forms an alloy-like acceptor phase, so that the energy levels, absorption and packing can be tuned continuously without disturbing the morphology. For example, the asymmetric guest acceptor Z8 with phenyl-substituted alkyl side chains, added to D18:L8-BO, acted as an alloy acceptor and delivered a PCE of 20.2% (certified 19.8%). DFT and MD calculations showed that its asymmetric structure delocalizes the excitons, which reduces the non-radiative energy loss and charge recombination, and that its side chains improve the intermolecular packing.^187^ Similar ternary strategies based on a DFT-designed guest acceptor (*o*-BTP-eC9) and a halogenated polymer donor (PBTQ-Cl) have been discussed in Section 8.3.^92,181^ For such multicomponent blends, the computational challenge moves from single molecules to mixtures. The miscibility of the guest with the host, the distribution of energy levels in the alloyed phase and the energy transfer between the components can only be described by combining MD-generated morphologies with the quantum-chemical methods described in Sections 3 and 6.

## 9. Computational insights into NFA degradation and stability

Achieving long-term operational stability remains a critical bottleneck for the commercialization of NFA-based OSCs. The degradation of these devices results from a combination of photochemical, triplet-mediated, oxidative and morphological processes.^45^ Experimental techniques can identify the degradation products and the loss in device performance. However, the elementary steps behind them, *e.g.* conformational changes in the excited state, the activation of specific chemical bonds, the formation of triplet excitons and the diffusion of molecules, occur at the atomistic level. Computational methods, particularly DFT, TD-DFT and MD simulations, can therefore provide valuable insight into these processes and help to design more stable materials. In this section, the main degradation pathways of NFAs are discussed with the help of case studies, and the computational descriptors which can be used to predict them are identified, as summarized in Table 5.

**Table 5:** Main degradation pathways of NFA-based OSCs and the computational descriptors used to predict them

Degradation pathway	Mechanism	Computational descriptors	Methods	Mitigation strategy	Ref.
Photochemical	Photoisomerization and photo-oxidation of exocyclic vinyl linkages; conformational twisting	Torsional energy profile in S_0_/S_1_; C=C bond order upon excitation; ^1^O_2_ addition barrier; packing density	DFT, TD-DFT, MD	Planar non-fused backbones; outward side chains; dense packing	188 and 189
Triplet-mediated	Back charge transfer from ^3^CT to T_1_; singlet-oxygen sensitization	T_1_ energy; singlet–triplet gap; spin–orbit coupling; ^3^CT–T_1_ offset and coupling	TD-DFT, quantum chemistry	Hybridization of T_1_ and ^3^CT states	41 and 190
Oxidative	Electron or energy transfer to O_2_; radical attack	IP, EA, spin density, reactive sites	DFT (tuned RSH)	Deep frontier levels; halogenation of end groups	45
Thermal/morphological	Demixing, crystallization and diffusion of the acceptor	*χ*, *T*_g_, diffusion coefficient, dipole moment	MD, DFT	Controlled miscibility; high-*T*_g_ components; low-dipole donors	181 and 191

### 9.1 Photochemical degradation of the conjugated backbone

The exocyclic vinyl (C=C) linkages which connect the fused core to the end groups in A–D–A acceptors have been identified as the weakest points under illumination. A comparative study of fused, semi-fused and non-fused acceptors showed that the photoisomerization of these vinyl groups is a critical precursor to photodegradation, and it is followed by photo-oxidation leading to epoxide species. The non-fused acceptor PTIC degraded with a rate of 9.00 × 10^−4^ h^−1^, about 16 times slower than IT-4F (1.48 × 10^−2^ h^−1^), and the PTIC-based devices retained about 72% of their initial PCE after 500 h of illumination. The outward hexyl chains and the planar, sp^3^-carbon-free backbone of PTIC gave dense molecular packing (π–π distance of 3.369 Å), which provided a structural confinement that suppressed the rearrangement of the vinyl bonds.^188^ Similarly, a comparison of the planar O-IDTBR and the twisted O-IDFBR acceptors showed that photodegradation proceeds in three stages: photo-induced conformational change, then photo-oxidation and fragmentation, and finally complete bleaching of the chromophore. The already twisted molecule was more susceptible to degradation, which showed that the initial conformational change is a prerequisite for further degradation.^189^

These studies point to several quantities which can be computed before synthesis. DFT and TD-DFT can provide the potential energy surface for twisting of the end groups relative to the core in the ground and excited states, the change in bond order of the vinyl linkage upon excitation, and the energy barriers for addition of singlet oxygen across the C=C bond. MD simulations, on the other hand, can estimate the packing density and free volume around the vinyl linkage in the solid state, which determine the degree of structural confinement.

### 9.2 Triplet-mediated and oxidative degradation

Triplet excitons play an important role in both the efficiency and the stability of NFA-based devices. It has been found that in the benchmark PM6:Y6 blend, up to 90% of the non-geminate recombination proceeds through the formation of triplet excitons on the NFA, which reduces the Voc by 60 mV. Quantum-chemical calculations showed that engineering a strong hybridization between the NFA triplet exciton (T_1_) and the triplet charge-transfer state (^3^CT) can reduce the rate of back charge transfer from ^3^CT to T_1_ by an order of magnitude.^190^ Since long-lived triplet excitons can transfer energy to molecular oxygen and generate highly reactive singlet oxygen, the suppression of triplet formation is beneficial for photostability as well.^41^ In the presence of oxygen and moisture, the NFA can also transfer an electron to oxygen and form superoxide radicals. In this regard, DFT calculations with optimally tuned functionals are useful to determine the IPs and EAs, the spin density distribution and the reactive sites of the molecules. For instance, fluorination or chlorination of the end groups downshifts the frontier orbital levels and can reduce the chemical reactivity of the NFA towards environmental oxidants.^45^ The computational descriptors for these pathways include the T_1_ energy, the singlet–triplet gap, the spin–orbit coupling, and the energetic offset and electronic coupling between the ^3^CT and T_1_ states.

### 9.3 Thermal and morphological degradation

Under thermal stress, the donor:NFA blend can undergo phase separation, crystallization or excessive aggregation, which reduces the interfacial area and the efficiency of charge generation. A molecular interaction–diffusion framework has been proposed to predict this type of degradation. It combines the Flory–Huggins interaction parameter (*χ*), obtained from simulated phase diagrams, with the glass transition temperature (*T*_g_) and the activation energy (*E*_a_) for diffusion of the acceptor into the donor polymer. It has been observed that the diffusion of NFAs follows Arrhenius behaviour, and *E*_a_ scales linearly with the enthalpic interaction parameter. As a result, blends which are thermodynamically less miscible (higher *χ*) can be kinetically more stable.^191^ MD simulations are well suited to provide such parameters: the mixing energies and cohesive energy densities give *χ*, the density–temperature relation gives *T*_g_, and the mean square displacement gives the diffusion coefficients.

A recent example shows how DFT-based design can guide the development of stable devices. In a series of halogenated wide-bandgap polymer donors, DFT calculations showed that the dipole moment of the repeating unit follows the order PBTQ-Cl (3.91 D) < PBTQ-F (4.84 D) < PBTQ-2F (5.02 D). The lowest dipole moment of PBTQ-Cl was linked with better miscibility with the BTP-eC9 acceptor and suppressed large-scale phase separation. Accordingly, the PBTQ-Cl:BTP-eC9 device retained 87% of its initial PCE after 5700 h at 85 °C (*T*_80_ > 11 000 h), and the ternary device showed *T*_80_ > 4000 h at 85 °C with over 90% retention after 600 h of maximum-power-point tracking.^181^

### 9.4 Outlook: towards multiscale modelling of degradation

Most computational studies of NFA stability still consider isolated molecules or idealized blends. A realistic description of degradation under operational light and thermal stress requires multiscale approaches. For instance, interface morphologies generated by MD simulations can be used in quantum mechanics/molecular mechanics (QM/MM) calculations of the excited states and reaction barriers of NFAs in contact with oxygen, water or interlayer materials. Moreover, reactive force fields and machine-learning potentials of first-principles quality^192^ can follow bond-breaking processes over much longer time scales than DFT. Machine-learning models trained on accelerated-ageing data can also help to identify the molecular features which control device lifetime. The integration of these approaches is expected to make the prediction of stability as routine as the prediction of efficiency in the design of new NFAs.

## 10. Conclusion

Computational methods have become indispensable in the design of NFAs, and the combination of DFT, MD simulations and ML now covers the molecular, morphological and device-level aspects of NFA-based organic solar cells. This review shows that the reliability of DFT predictions depends primarily on the choice of the exchange–correlation functional. Semi-local functionals are not suitable for the electronic structure of large π-conjugated NFAs because of the SIE and the absence of dispersion. Global hybrids such as B3LYP remain useful for geometries and for trends within a series of related molecules. However, quantitative prediction of fundamental gaps, energy levels and charge-transfer excitations requires optimally tuned RSHs, and the screened variants of these functionals are needed for comparison with solid-state measurements. MD simulations provide the link between molecular structure and film morphology, *e.g.* π–π stacking, electronic coupling, miscibility and diffusion, whereas ML models have enabled the rapid screening of large chemical spaces and the generative design of new acceptors. At the same time, the performance of ML is still limited by the scarcity of high-quality data and the difficulty of predicting materials that lie outside the training set.

The stability of NFA-based devices has now become as important as their efficiency. The computational insight discussed in this review shows that photoisomerization and photo-oxidation of the vinyl linkages, triplet-mediated processes and morphological demixing are the main degradation pathways, and that each of them can be linked with computable descriptors, such as torsional energy profiles, triplet energies and ^3^CT–T_1_ coupling, and interaction parameters and diffusion coefficients. Future progress is expected from the integration of these descriptors into multiscale workflows combining MD, QM/MM and machine-learning potentials, validated against accelerated-ageing experiments. Such a synergistic computational–experimental approach will help to design NFAs which combine high efficiency with long operational lifetime, and it will contribute towards the commercialization of organic photovoltaics.

## Conflicts of interest

The authors declare that they have no known competing financial interests or personal relationships that could have appeared to influence the work reported in this paper.

## Data Availability

No primary research results, software or code have been included and no new data were generated or analysed as part of this review.

## References

[cit1] Irfan M., Akash A., Sultan N., Janjua M. R. S. A. (2026). RSC Adv..

[cit2] Mahdi A. S., Shaker L. M., Alamiery A. (2024). J. Opt..

[cit3] Galdino Alves T. Y., Mota I. C., Marcate L. A., Vieira Marques M. D. F. (2026). RSC Adv..

[cit4] Wang L., Chen C., Fu Y., Guo C., Li D., Cheng J., Sun W., Gan Z., Sun Y., Zhou B. (2024). et al.. Nat. Energy.

[cit5] Jain A., Kothari R., Tyagi V., Rajamony R. K., Ahmad M. S., Singh H. M., Raina S., Pandey A. (2024). Sustainable Energy Technol. Assess..

[cit6] Asmare E., Bekri N., Nchinda L. T., Hone F. G., Mammo W., Krüger T. P., Tegegne N. A. (2024). J. Phys. Chem. C.

[cit7] Brédas J.-L., Norton J. E., Cornil J., Coropceanu V. (2009). Acc. Chem. Res..

[cit8] Rim S.-B., Fink R. F., Schöneboom J. C., Erk P., Peumans P. (2007). Appl. Phys. Lett..

[cit9] Sahu P. N., Vasarla A. D., Kumili R. K., Sen A. (2024). Comput. Theor. Chem..

[cit10] Sun R., Guo J., Sun C., Wang T., Luo Z., Zhang Z., Jiao X., Tang W., Yang C., Li Y. (2019). et al.. Energy Environ. Sci..

[cit11] Mahariq I., Sharma R., Kumar A., Vaghela K., Rekha M. M., Verma L., Kumar M. R., Shallan M. A., Almehizia A. A. (2025). J. Phys. Chem. Solids.

[cit12] Raheem A. A., Praveen C. (2024). J. Mater. Chem. C.

[cit13] Tong Y., Xiao Z., Du X., Zuo C., Li Y., Lv M., Yuan Y., Yi C., Hao F., Hua Y. (2020). et al.. Sci. China: Chem..

[cit14] He Y., Li Y. (2011). Phys. Chem. Chem. Phys..

[cit15] Zhang C., Yu R., Lv Q., Li S., Yuan H., Huang B., Tan Z. (2025). ChemSusChem.

[cit16] He Y., Chen H.-Y., Hou J., Li Y. (2010). J. Am. Chem. Soc..

[cit17] Liu M., Zhu P., Lai S., Zhang X., Fu Y., Xue L., Liu D., Feng K., Lu X., Guo X. (2025). et al.. Chin. J. Chem..

[cit18] Zhang Z., Wang Y., Sun C., Liu Z., Wang H., Xue L., Zhang Z.-G. (2022). Nano Sel..

[cit19] Kupgan G., Chen X.-K., Brédas J.-L. (2019). ACS Mater. Lett..

[cit20] Zhang G., Zhao J., Chow P. C. Y., Jiang K., Zhang J., Zhu Z., Zhang J., Huang F., Yan H. (2018). Chem. Rev..

[cit21] Kamalinahad S., Vessally E., Noormohammadbeigi M. (2025). J. Photochem. Photobiol., A.

[cit22] Chen M., Liu J., Song P., Li Y. (2025). J. Mol. Struct..

[cit23] Dominguez R., Moreno L. M., Langa F., de la Cruz P. (2024). Curr. Opin. Colloid Interface Sci..

[cit24] Cheng P., Li G., Zhan X., Yang Y. (2018). Nat. Photonics.

[cit25] Zhan X., Tan Z., Domercq B., An Z., Zhang X., Barlow S., Li Y., Zhu D., Kippelen B., Marder S. R. (2007). J. Am. Chem. Soc..

[cit26] Lin Y., He Q., Zhao F., Huo L., Mai J., Lu X., Su C.-J., Li T., Wang J., Zhu J., Sun Y., Wang C., Zhan X. (2016). J. Am. Chem. Soc..

[cit27] Lin Y., Wang J., Zhang Z.-G., Bai H., Li Y., Zhu D., Zhan X. (2015). Adv. Mater..

[cit28] Wen Z.-C., Yin H., Hao X.-T. (2021). Surf. Interfaces.

[cit29] Wu Q., Ding S., Sun A., Xia Y. (2024). Mater. Today Chem..

[cit30] Shahzadi S., Shahzadi T., Shafiq Z., Janjua M. R. S. A. (2024). High Energy Chem..

[cit31] Zhang X.-X., Yu X.-F., Xiao B. (2023). J. Phys. Chem. A.

[cit32] Xiang Y., Xu C., Zheng S. (2023). Int. J. Mol. Sci..

[cit33] Wu H., Ma Z., Li M., Lu H., Tang A., Zhou E., Wen J., Sun Y., Tress W., Olsen J. M. H. (2023). et al.. Energy Environ. Sci..

[cit34] Irfan A., Mahmood A., Al-Sehemi A. G., Ahmad F. (2019). J. Mol. Struct..

[cit35] Fu Y., Lee T. H., Chin Y.-C., Pacalaj R. A., Labanti C., Park S. Y., Dong Y., Cho H. W., Kim J. Y., Minami D. (2023). et al.. Nat. Commun..

[cit36] Chen K., Wei H., Chen P.-A., Liu Y., Guo J., Xia J., Xie H., Qiu X., Hu Y. (2022). Front. Optoelectron..

[cit37] Lee M.-H. (2024). Sol. Energy.

[cit38] Ahmed D. R., Muhammadsharif F. F. (2025). Processes.

[cit39] Wei Q., Liu W., Leclerc M., Yuan J., Chen H., Zou Y. (2020). Sci. China:Chem..

[cit40] Ravele T., Fuku X. G., Kebede M. A. (2025). Energy Fuels.

[cit41] Zhou R., Gao X., Chen H., Gao J., Liu Z. (2025). J. Mater. Chem. A.

[cit42] Yan C., Barlow S., Wang Z., Yan H., Jen A. K.-Y., Marder S. R., Zhan X. (2018). Nat. Rev. Mater..

[cit43] Wang J., Xue P., Jiang Y., Huo Y., Zhan X. (2022). Nat. Rev. Chem..

[cit44] Wang J., Xie Y., Chen K., Wu H., Hodgkiss J. M., Zhan X. (2024). Nat. Rev. Phys..

[cit45] Cheng P., Zhan X. (2016). Chem. Soc. Rev..

[cit46] Han J.-H., Zhao Z.-W., Pan Q.-Q., Wang L.-L., Zhou H.-P., Su Z.-M. (2025). ACS Appl. Mater. Interfaces.

[cit47] Yuan J., Zhang Y., Zhou L., Zhang G., Yip H.-L., Lau T.-K., Lu X., Zhu C., Peng H., Johnson P. A., Leclerc M., Cao Y., Ulanski J., Li Y., Zou Y. (2019). Joule.

[cit48] Yuan J., Huang T., Cheng P., Zou Y., Zhang H., Yang J. L., Chang S.-Y., Zhang Z., Huang W., Wang R., Meng D., Gao F., Yang Y. (2019). Nat. Commun..

[cit49] Zhao J., Yao C., Ali M. U., Miao J., Meng H. (2020). Mater. Chem. Front..

[cit50] Yao H., Ye L., Zhang H., Li S., Zhang S., Hou J. (2016). Chem. Rev..

[cit51] Liu Y., Zhang C., Hao D., Zhang Z., Wu L., Li M., Feng S., Xu X., Liu F., Chen X. (2018). et al.. Chem. Mater..

[cit52] Yuan J., Zou Y. (2022). Org. Electron..

[cit53] Yang Y. (2021). ACS Nano.

[cit54] Ziffer M. E., Jo S. B., Zhong H., Ye L., Liu H., Lin F., Zhang J., Li X., Ade H. W., Jen A. K.-Y. (2018). et al.. J. Am. Chem. Soc..

[cit55] Luo D., Brabec C. J., Kyaw A. K. K. (2023). Nano Energy.

[cit56] Yang J., Li Q., Li Z. (2024). Sci. China Mater..

[cit57] Wang P., Li Y., Han C., Wang J., Bi F., Zheng N., Yang J., Wang J., Bao X. (2022). J. Mater. Chem. A.

[cit58] Bauer N., Zhang Q., Rech J. J., Dai S., Peng Z., Ade H., Wang J., Zhan X., You W. (2019). Nano Res..

[cit59] Shoaee S., Luong H. M., Song J., Zou Y., Nguyen T., Neher D. (2024). Adv. Mater..

[cit60] Hu Y., Wang J., Yan C., Cheng P. (2022). Nat. Rev. Mater..

[cit61] Tao M., Peng L., Gu F. L. (2022). New J. Chem..

[cit62] Li X., Zhang Q., Yu J., Xu Y., Zhang R., Wang C., Zhang H., Fabiano S., Liu X., Hou J. (2022). et al.. Nat. Commun..

[cit63] Menke S. M., Ran N. A., Bazan G. C., Friend R. H. (2018). Joule.

[cit64] Sun H., Chen F., Chen Z.-K. (2019). Mater. Today.

[cit65] Liu F., Zhou Z., Zhang C., Vergote T., Fan H., Liu F., Zhu X. (2016). J. Am. Chem. Soc..

[cit66] Qian D., Zheng Z., Yao H., Tress W., Hopper T. R., Chen S., Li S., Liu J., Chen S., Zhang J. (2018). et al.. Nat. Mater..

[cit67] Wang P., Bi F., Li Y., Han C., Zheng N., Zhang S., Wang J., Wu Y., Bao X. (2022). Adv. Funct. Mater..

[cit68] Mohan O., Choksi T. S., Lapkin A. A. (2024). Catal. Sci. Technol..

[cit69] Shen H., Ren Y., Li J., Xu Y., Han C., Zou W., Xu H., Sun Y., Kan Y., Gao K. (2023). et al.. Chin. J. Chem..

[cit70] Hohenberg P., Kohn W. (1964). Phys. Rev..

[cit71] Schwarz K., Sham L. J., Mattsson A. E., Scheffler M. (2016). Computation.

[cit72] Ziesche P., Kurth S., Perdew J. P. (1998). Comput. Mater. Sci..

[cit73] Langreth D. C., Mehl M. J. (1983). Phys. Rev. B:Condens. Matter Mater. Phys..

[cit74] Simón L., Goodman J. M. (2011). Org. Biomol. Chem..

[cit75] Jaffe J. E., Snyder J. A., Lin Z., Hess A. C. (2000). Phys. Rev. B:Condens. Matter Mater. Phys..

[cit76] Mao G.-Q., Yan Z.-Y., Xue K.-H., Ai Z., Yang S., Cui H., Yuan J.-H., Ren T.-L., Miao X. (2022). J. Phys.: Condens. Matter.

[cit77] Li D., Zhang X., Liu D., Wang T. (2020). J. Mater. Chem. A.

[cit78] Franco L. R., Marchiori C., Araujo C. M. (2023). J. Chem. Phys..

[cit79] Jaramillo J., Scuseria G. E., Ernzerhof M. (2003). J. Chem. Phys..

[cit80] Pourebrahimi S., Pirooz M. (2024). Sol. Energy.

[cit81] Ans M., Iqbal J., Eliasson B., Saif M. J., Ayub K. (2019). Comput. Mater. Sci..

[cit82] Kanwal N., Hussain R., Sattar A., Assiri M. A., Imran M., Hussain A., Yawer M. A., Hussain R., Mehboob M. Y., Khalid M. (2022). et al.. Opt. Quantum Electron..

[cit83] Ali A., Farid T., Rafiq M. I., Zhou B., Tang W. (2022). Phys. Chem. Chem. Phys..

[cit84] Chanana G., Batra K. (2021). J. Mol. Model..

[cit85] Sarkar R., Boggio-Pasqua M., Loos P.-F., Jacquemin D. (2021). J. Chem. Theory Comput..

[cit86] Krukau A. V., Scuseria G. E., Perdew J. P., Savin A. (2008). J. Chem. Phys..

[cit87] Patel K., Khatua R., Patrikar K., Mondal A. (2024). J. Chem. Phys..

[cit88] Refaely-Abramson S., Sharifzadeh S., Jain M., Baer R., Neaton J. B., Kronik L. (2013). Phys. Rev. B:Condens. Matter Mater. Phys..

[cit89] Bhandari S., Cheung M. S., Geva E., Kronik L., Dunietz B. D. (2018). J. Chem. Theory Comput..

[cit90] Guo Q., Guo Q., Geng Y., Tang A., Zhang M., Du M., Sun X., Zhou E. (2021). Mater. Chem. Front..

[cit91] Wei Y., Chen Z., Lu G., Yu N., Li C., Gao J., Gu X., Hao X., Lu G., Tang Z. (2022). et al.. Adv. Mater..

[cit92] Fu J., Yang Q., Huang P., Chung S., Cho K., Kan Z., Liu H., Lu X., Lang Y., Lai H. (2024). et al.. Nat. Commun..

[cit93] Li C., Song J., Lai H., Zhang H., Zhou R., Xu J., Huang H., Liu L., Gao J., Li Y. (2025). et al.. Nat. Mater..

[cit94] Zhang M., Guo X., Ma W., Ade H., Hou J. (2015). Adv. Mater..

[cit95] Lu H., Liu W., Ran G., Liang Z., Li H., Wei N., Wu H., Ma Z., Liu Y., Zhang W. (2023). et al.. Angew. Chem..

[cit96] Cha H., Zheng Y., Dong Y., Lee H. H., Wu J., Bristow H., Zhang J., Lee H. K. H., Tsoi W. C., Bakulin A. A. (2020). et al.. Adv. Energy Mater..

[cit97] Pan Q.-Q., Li S.-B., Wu Y., Geng Y., Zhang M., Su Z.-M. (2018). Org. Electron..

[cit98] Huo Y., Zhang H.-L., Zhan X. (2019). ACS Energy Lett..

[cit99] Naeem N., Tahir T., Ans M., Rasool A., Shehzad R. A., Iqbal J. (2021). Comput. Theor. Chem..

[cit100] Wang K., Zhao F., Zhu Y., He Y., Liu Z., Han X., Ai Q., Shen X., Li B., Zhang J. (2024). et al.. J. Mater. Chem. A.

[cit101] Chung H. Y., Park J.-H., Cui J., Kim S.-Y., Oh J., Kim D., Park S. Y. (2021). J. Phys. Chem. C.

[cit102] Zhang Z., Li L., Xu C., Jin P., Huang M., Li Y., Wang H., Yi Y., Zhang C., Yang Y. (2022). et al.. Cell Rep. Phys. Sci..

[cit103] Coughlin J. E., Henson Z. B., Welch G. C., Bazan G. C. (2014). Acc. Chem. Res..

[cit104] Demeter D., Rousseau T., Leriche P., Cauchy T., Po R., Roncali J. (2011). Adv. Funct. Mater..

[cit105] Wang J., Jiang X., Wu H., Feng G., Wu H., Li J., Yi Y., Feng X., Ma Z., Li W. (2021). et al.. Nat. Commun..

[cit106] Mishra A., Popovic D., Vogt A., Kast H., Leitner T., Walzer K., Pfeiffer M., Mena-Osteritz E., Bäuerle P. (2014). Adv. Mater..

[cit107] Li S., Li C.-Z., Shi M., Chen H. (2020). ACS Energy Lett..

[cit108] Körzdörfer T., Sears J. S., Sutton C., Brédas J.-L. (2011). J. Chem. Phys..

[cit109] Capelle K., Vignale G., Ullrich C. A. (2010). Phys. Rev. B:Condens. Matter Mater. Phys..

[cit110] Sabuj M. A., Huda M. M., Sarap C. S., Rai N. (2021). Mater. Adv..

[cit111] Körzdörfer T., Brédas J.-L. (2014). Acc. Chem. Res..

[cit112] Zhugayevych A., Lin K.-H., Andrienko D. (2023). J. Chem. Phys..

[cit113] Hasenburg F. H., Lin K.-H., van der Zee B., Blom P. W. M., Andrienko D., Wetzelaer G.-J. A. H. (2023). APL Mater..

[cit114] Ye C., Wang Y., Bi Z., Guo X., Fan Q., Chen J., Ou X., Ma W., Zhang M. (2018). Org. Electron..

[cit115] Manzoor F., Iqbal J., Zara Z., Eliasson B., Mahr M. S., Ayub K. (2018). ChemistrySelect.

[cit116] Guégano X., Kanibolotsky A. L., Blum C., Mertens S. F. L., Liu S.-X., Neels A., Hagemann H., Skabara P. J., Leutwyler S., Wandlowski T. (2009). et al.. Chem. – Eur. J..

[cit117] Taherpour A. A., Jamshidi M., Rezaei O. (2017). J. Mol. Graphics Modell..

[cit118] Taherpour A. A., Jamshidi M., Rezaei O. (2017). J. Mol. Struct..

[cit119] Komjáti B., Urai Á., Hosztafi S., Kökösi J., Kováts B., Nagy J., Horváth P. (2016). Spectrochim. Acta, Part A.

[cit120] Lan J.-L., Liu X.-N., Xiao C.-N., Sui M.-Y., Sun G.-Y. (2024). J. Photochem. Photobiol., A.

[cit121] Refaely-Abramson S., Sharifzadeh S., Govind N., Autschbach J., Neaton J. B., Baer R., Kronik L. (2012). Phys. Rev. Lett..

[cit122] Wang J., Zhu J., Li C., Lin Y., Yang Y., Ma Z., Lu Y. (2023). Adv. Funct. Mater..

[cit123] Kronik L., Neaton J. B. (2016). Annu. Rev. Phys. Chem..

[cit124] Kronik L., Stein T., Refaely-Abramson S., Baer R. (2012). J. Chem. Theory Comput..

[cit125] Lu Q., Qiu M., Zhao M., Li Z., Li Y. (2019). Polymers.

[cit126] Farhat A., Khera R. A., Iqbal S., Iqbal J. (2020). Opt. Mater..

[cit127] Akram S. J., Hadia N. M. A., Shawky A. M., Iqbal J., Khan M. I., Alatawi N. S., Ibrahim M. A. A., Ans M., Khera R. A. (2023). ACS Omega.

[cit128] Zhang J., Liu W., Zhang M., Liu Y., Zhou G., Xu S., Zhang F., Zhu H., Liu F., Zhu X. (2019). iScience.

[cit129] Halaby S., Martynowycz M. W., Zhu Z., Tretiak S., Zhugayevych A., Gonen T., Seifrid M. (2021). Chem. Mater..

[cit130] Li Y., Huang W., Zhao D., Wang L., Jiao Z., Huang Q., Wang P., Sun M., Yuan G. (2022). Molecules.

[cit131] Yoon S., Shin E.-Y., Cho N.-K., Park S., Woo H. Y., Son H. J. (2021). J. Mater. Chem. A.

[cit132] Unke O. T., Chmiela S., Sauceda H. E., Gastegger M., Poltavsky I., Schütt K. T., Tkatchenko A., Müller K.-R. (2021). Chem. Rev..

[cit133] Baumeier B., Kirkpatrick J., Andrienko D. (2010). Phys. Chem. Chem. Phys..

[cit134] Rühle V., Lukyanov A., May F., Schrader M., Vehoff T., Kirkpatrick J., Baumeier B., Andrienko D. (2011). J. Chem. Theory Comput..

[cit135] Marcus R. A. (1993). Rev. Mod. Phys..

[cit136] Coropceanu V., Cornil J., da Silva Filho D. A., Olivier Y., Silbey R., Brédas J.-L. (2007). Chem. Rev..

[cit137] Zhang G., Chen X.-K., Xiao J., Chow P. C. Y., Ren M., Kupgan G., Jiao X., Chan C. C. S., Du X., Xia R. (2020). et al.. Nat. Commun..

[cit138] ul Ain Q., Akram W., Yaqoob H., Shahid M., Majid A., Altaf M., Nielsen C. B., Ashraf R. S. (2026). Adv. Electron. Mater..

[cit139] Riley D. B., Meredith P., Armin A., Sandberg O. J. (2022). J. Phys. Chem. Lett..

[cit140] Pelzer K. M., Vázquez-Mayagoitia Á., Ratcliff L. E., Tretiak S., Bair R. A., Gray S. K., Van Voorhis T., Larsen R. E., Darling S. B. (2017). Chem. Sci..

[cit141] Li C., Zhou J., Song J., Xu J., Zhang H., Zhang X., Guo J., Zhu L., Wei D., Han G. (2021). et al.. Nat. Energy.

[cit142] Setsoafia D. D. Y., Ram K. S., Mehdizadeh-Rad H., Ompong D., Singh J. (2024). Energies.

[cit143] Raschka S., Patterson J., Nolet C. (2020). Information.

[cit144] Siddiqui H., Usmani T. (2024). ACS Omega.

[cit145] Sun W., Zheng Y., Yang K., Zhang Q., Shah A. A., Wu Z., Sun Y., Feng L., Chen D., Xiao Z. (2019). et al.. Sci. Adv..

[cit146] Zhao Z., Geng Y., Troisi A., Ma H. (2022). Adv. Intell. Syst..

[cit147] Wu Y., Guo J., Sun R., Min J. (2020). npj Comput. Mater..

[cit148] Malhotra P., Khandelwal K., Biswas S., Chen F.-C., Sharma G. D. (2022). J. Mater. Chem. C.

[cit149] Greenstein B. L. , PhD thesis, University of Pittsburgh, 2024

[cit150] Jiang Y., Yao C., Yang Y., Wang J. (2024). Sol. RRL.

[cit151] Tang H., Bai Y., Zhao H., Qin X., Hu Z., Zhou C., Huang F., Cao Y. (2024). Adv. Mater..

[cit152] Lee J., Ko S., Seifrid M., Lee H., McDowell C., Luginbuhl B. R., Karki A., Cho K., Nguyen T.-Q., Bazan G. C. (2018). Adv. Energy Mater..

[cit153] Saeed M. U., Iqbal J., Mehmood R. F., Riaz M., Akram S. J., Somaily H., Shawky A. M., Raheel M., Khan M. I., Rashid E. U. (2022). et al.. J. Phys. Chem. Solids.

[cit154] Tufail M. K., Shah S. S. A., Khan S., Ahmad F., Kiruri L. W., Abbasi M. S., Ahmad A. (2024). Chem. Phys. Lett..

[cit155] Rodríguez-Martínez X., Pascual-San-José E., Campoy-Quiles M. (2021). Energy Environ. Sci..

[cit156] Ayub A. R., Akram W., Yaqoob U., Salba, Maqsood N., Rafiq S., Nabat K. Y., Anwer A., Somaily H. H., Alansari A. (2025). et al.. Spectrochim. Acta, Part A.

[cit157] Sahu H., Rao W., Troisi A., Ma H. (2018). Adv. Energy Mater..

[cit158] Nagasawa S., Al-Naamani E., Saeki A. (2018). J. Phys. Chem. Lett..

[cit159] Lopez S. A., Pyzer-Knapp E. O., Simm G. N., Lutzow T., Li K., Seress L. R., Hachmann J., Aspuru-Guzik A. (2016). Sci. Data.

[cit160] Moineau-Chane Ching K. I. (2023). Energies.

[cit161] Menon D., Ranganathan R. (2022). ACS Omega.

[cit162] Torimtubun A. A. A., Alonso-Navarro M. J., Quesada-Ramírez A., Rodríguez-Martínez X., Segura J. L., Goñi A. R., Campoy-Quiles M. (2024). Sol. RRL.

[cit163] Qundeel, Adnan M., Hussain R., Shehzad R. A., Muhammad S., Mustafa G., Irshad Z. (2024). Int. J. Quantum Chem..

[cit164] Zoromba M. S., Maddah H., Abdel-Aziz M., Al-Hossainy A. F. (2022). J. Ind. Eng. Chem..

[cit165] Adamo C., Jacquemin D. (2013). Chem. Soc. Rev..

[cit166] Yan J., Rodríguez-Martínez X., Pearce D., Douglas H., Bili D., Azzouzi M., Eisner F., Virbule A., Rezasoltani E., Belova V. (2022). et al.. Energy Environ. Sci..

[cit167] Lee M.-H. (2022). Sol. Energy.

[cit168] Choudhary K., Bercx M., Jiang J., Pachter R., Lamoen D., Tavazza F. (2019). Chem. Mater..

[cit169] Liu X., Shao Y., Lu T., Chang D., Li M., Lu W. (2022). Mater. Des..

[cit170] Hao T., Leng S., Yang Y., Zhong W., Zhang M., Zhu L., Song J., Xu J., Zhou G., Zou Y. (2021). et al.. Patterns.

[cit171] Tan J. D., Ramalingam B., Chellappan V., Gupta N. K., Dillard L., Khan S. A., Galvin C., Hippalgaonkar K. (2024). ACS Energy Lett..

[cit172] Li G., Feng L.-W., Mukherjee S., Jones L. O., Jacobberger R. M., Huang W., Young R. M., Pankow R. M., Zhu W., Lu N. (2022). et al.. Energy Environ. Sci..

[cit173] Lee M.-H. (2019). Adv. Electron. Mater..

[cit174] Zhang Y., Lang Y., Li G. (2023). EcoMat.

[cit175] Zhu L., Zhang M., Zhong W., Leng S., Zhou G., Zou Y., Su X., Ding H., Gu P., Liu F. (2021). et al.. Energy Environ. Sci..

[cit176] Zhao W., Qian D., Zhang S., Li S., Inganäs O., Gao F., Hou J. (2016). Adv. Mater..

[cit177] Romo-Gutiérrez A., Cisneros-García Z., Rodríguez-Zavala J. (2022). Mol. Phys..

[cit178] Cisneros-García Z., Guirado-López R., Rodríguez-Zavala J., Facio-Muñoz J. (2024). Comput. Theor. Chem..

[cit179] Han G., Yi Y. (2022). Acc. Chem. Res..

[cit180] Dahiya H., Suthar R., Khandelwal K., Karak S., Sharma G. D. (2022). ACS Appl. Electron. Mater..

[cit181] Gao J., Hu H., Li X., Lin C., Liao X., Li F., Xie J., Yang M., Peng R., Ge Z. (2026). Adv. Energy Mater..

[cit182] Wei N., Chen J., Shang B., Xie Q., Song H., Zhang W., Hu H., Zhang J., Lu H., Hao B. (2026). et al.. Adv. Funct. Mater..

[cit183] Fan Q., An Q., Lin Y., Xia Y., Li Q., Zhang M., Su W., Peng W., Zhang C., Liu F. (2020). et al.. Energy Environ. Sci..

[cit184] Wu Y., Yuan Y., Sorbelli D., Cheng C., Michalek L., Cheng H.-W., Jindal V., Zhang S., LeCroy G., Gomez E. D. (2024). et al.. Nat. Commun..

[cit185] Wang Z., Guo Y., Liu X., Shu W., Han G., Ding K., Mukherjee S., Zhang N., Yip H.-L., Yi Y. (2024). et al.. Nat. Commun..

[cit186] Wang J., Sun C., Li Y., Bi F., Jiang H., Yang C., Bao X., Chu J. (2025). Nat. Commun..

[cit187] Jiang Y., Sun S., Xu R., Liu F., Miao X., Ran G., Liu K., Yi Y., Zhang W., Zhu X. (2024). Nat. Energy.

[cit188] Liu Z.-X., Yu Z.-P., Shen Z., He C., Lau T.-K., Chen Z., Zhu H., Lu X., Xie Z., Chen H., Li C.-Z. (2021). Nat. Commun..

[cit189] Luke J., Speller E. M., Wadsworth A., Wyatt M. F., Dimitrov S., Lee H. K. H., Li Z., Tsoi W. C., McCulloch I., Bagnis D. (2019). et al.. Adv. Energy Mater..

[cit190] Gillett A. J., Privitera A., Dilmurat R., Karki A., Qian D., Pershin A., Londi G., Myers W. K., Lee J., Yuan J. (2021). et al.. Nature.

[cit191] Ghasemi M., Balar N., Peng Z., Hu H., Qin Y., Kim T., Rech J. J., Bidwell M., Mask W., McCulloch I., You W., Amassian A., Risko C., O'Connor B. T., Ade H. (2021). Nat. Mater..

[cit192] Jinnouchi R., Karsai F., Kresse G. (2025). Chem. Sci..

